# Multi-stage mechanisms of tumor metastasis and therapeutic strategies

**DOI:** 10.1038/s41392-024-01955-5

**Published:** 2024-10-11

**Authors:** Zaoqu Liu, Jingqi Chen, Yuqing Ren, Shutong Liu, Yuhao Ba, Anning Zuo, Peng Luo, Quan Cheng, Hui Xu, Xinwei Han

**Affiliations:** 1 Department of Interventional Radiology, The First Affiliated Hospital of Zhengzhou University, Zhengzhou, Henan China; 2 Interventional Institute of Zhengzhou University, Zhengzhou, Henan China; 3 Interventional Treatment and Clinical Research Center of Henan Province, Zhengzhou, Henan China; 4 Institute of Basic Medical Sciences, Chinese Academy of Medical Sciences and Peking Union Medical College, Beijing, China; 5 Department of Clinical Medicine, Zhengzhou University, Zhengzhou, Henan China; 6 Department of Respiratory and Critical Care Medicine, The First Affiliated Hospital of Zhengzhou University, Zhengzhou, Henan China; 7 The Department of Oncology, Zhujiang Hospital, Southern Medical University, Guangzhou, China; 8 Department of Neurosurgery, Xiangya Hospital, Central South University, Changsha, China

**Keywords:** Cancer microenvironment, Cancer therapy, Metastasis

## Abstract

The cascade of metastasis in tumor cells, exhibiting organ-specific tendencies, may occur at numerous phases of the disease and progress under intense evolutionary pressures. Organ-specific metastasis relies on the formation of pre-metastatic niche (PMN), with diverse cell types and complex cell interactions contributing to this concept, adding a new dimension to the traditional metastasis cascade. Prior to metastatic dissemination, as orchestrators of PMN formation, primary tumor-derived extracellular vesicles prepare a fertile microenvironment for the settlement and colonization of circulating tumor cells at distant secondary sites, significantly impacting cancer progression and outcomes. Obviously, solely intervening in cancer metastatic sites passively after macrometastasis is often insufficient. Early prediction of metastasis and holistic, macro-level control represent the future directions in cancer therapy. This review emphasizes the dynamic and intricate systematic alterations that occur as cancer progresses, illustrates the immunological landscape of organ-specific PMN creation, and deepens understanding of treatment modalities pertinent to metastasis, thereby identifying some prognostic and predictive biomarkers favorable to early predict the occurrence of metastasis and design appropriate treatment combinations.

## Introduction

Cancer metastasis is a significant public health issue worldwide, characterized by its highly variable nature.^1^ Metastasis is the primary challenge for cancer patients, accounting for approximately 90% of cancer-related mortality.^2^ Targeting metastasis seeding and colonization remains an unresolved challenge despite extensive research available.^3^ Continued study of the biological mechanisms underlying tumor cell (TC) dissemination and outgrowth is essential.^4^

**Fig. 1:**
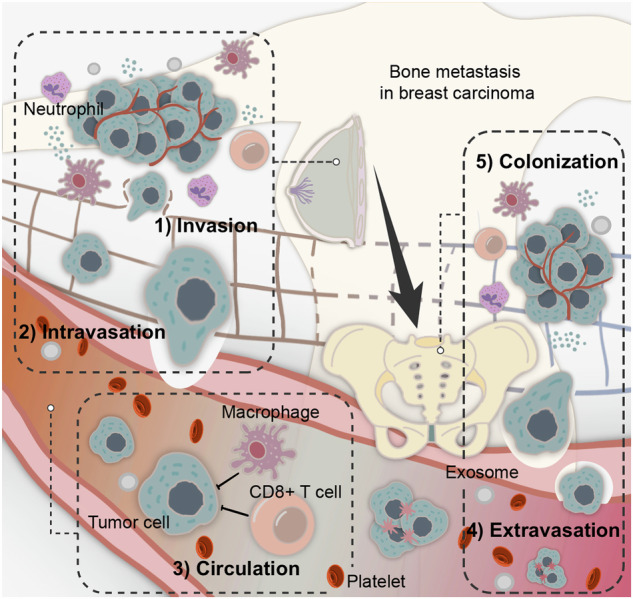
Overview of the metastatic cascade in bone metastasis. In patients with tumors, a large number of cancer cells are released in the circulation on a daily basis. The process of a portion of cancer cells moving away from the primary tumor site to form a secondary tumor at a secondary site is called “metastasis”. Cancer metastasis mainly includes five steps: invasion, intravasation, circulation, extravasation, and colonization. CTCs break through the basement membrane matrix enclosing the cancer nest, intravaste the surrounding blood vessels, and circulate in the blood, where they endure physical pressure and are attacked by immune cells. Upon reaching secondary sites, CTCs undergo extravasation and infiltrate secondary locations. Successful extravasation depends on interactions between cancer cells and endothelial cells. Subsequently, cancer cells adapt, and proliferate in the metastatic organ, gradually forming metastatic lesions. The ability of cancer cells to invade adjacent tissues and establish distant colonies is a hallmark of malignancy. Advances in understanding the various stages of tumor metastasis have revealed key molecular mechanisms, such as changes in cell adhesion molecules, EMT, and interactions with ECM components and immune cells within the microenvironment. Comprehensive knowledge of the multi-stage process of tumor metastasis from invasion to colonization is beneficial for identifying novel therapeutic targets and interventions aimed at disrupting metastatic progression and improving patient survival rates

TCs migrate throughout the lymphatic and blood circulations during the metastatic phase, leaving the primary site and eventually reaching distant areas, where they form visible macrometastases^5^ (Fig. 1). TCs gradually expand and invade surrounding tissues and stroma as they begin to form the primary tumor. At this point, “circulating tumor cells (CTCs)” is the term used to describe the TCs that have entered the bloodstream.^6^ Additionally, a growing number of studies have shown that the intrinsic processes of cancer cells alone do not fully account for the emergence of metastases due to the interconnections between cancer cells and their altered microenvironmental components, such as immunosurveillance.^7^ Therefore, only a limited number of CTCs with epithelial-mesenchymal transition (EMT) metastatic properties survive and infiltrate distant organs after they break away from the primary tumor and infiltrate into the bloodstream. These cancer cells enhance their metastatic potential through various methods, such as homotypic clustering and heterotypic interactions between immune and stromal cells. These mechanisms facilitate the formation of premetastatic niches, the successful colonization of other organs, and the development of secondary tumors.^6^ Thus, an important characteristic of cancer is its ability to evade immune destruction.^8^

The PMN is a microenvironment prepared for the lodging of CTCs in specific organs, consisting of unique resident cell types, extracellular matrix (ECM) components, and infiltrating cell populations. The variety of cell types and intricate interactions have conceptualized PMN.^9^ PMN manifests key attributes such as thrombosis, alterations in vascular permeability, ECM remodeling, and anomalous immunosuppressive inflammatory changes.^10^ The orchestration of organ-specific metastasis hinges on PMN formation, a process usually guided by extracellular vesicles (EVs), including microvesicles, exosomes, and large cancer vesicles released from malignant cells.^11^ Among them, exosomes from tumors may circulate in the bloodstream conveying inflammatory factors, PD-L1, and other compounds that might suppress the immune system, creating an immunosuppressive, inflammatory microenvironment favorable to the tumor in the pre-metastatic microenvironment. The DNA and coding or non-coding RNA fragments they carry are involved in directing the metastatic behavior of CTCs, while exosomal integrins may also interact with the ECM to promote subsequent metastatic colonization.^12^

Metastases frequently emerge years after the completion of local and systemic cancer therapies. This phenomenon strongly indicates that even after efficient cancer treatments, residual cancerous cells or minimal residual disease can persist in a dormant state. This dormancy allows the TCs to withstand physical assaults and evade immune surveillance. It is reported that CTCs enter a dormant state early in the metastatic process by undergoing phenotypic, genetic, and functional alterations.^13^ EMT, for example, is an inherent mechanism that regulates the metastatic dissemination of cancer cells.^7^ Despite tremendous advancements in cancer research, diagnosis, and treatment, the majority of patients with advanced metastatic illness remain incurable due to dormant mechanisms and medication resistance.^14^ There is a summary of potential drug resistance mechanisms existing in various stages of tumor progression in current tumor therapy. (Table 1).

**Table 1:** Summary of mechanisms of drug resistance emerging in current tumor treatments

Types		Molecules	Cancer types	Drugs	Mechanisms	Ref.
Genetically heterogeneity	Gene mutation	EGFR T790M ATP threonine methionine	non-small-cell lung cancer	gefitinib erlotinib	The **mutation of T790M** confers resistance, which may be pre-existing or may have been adaptively acquired by small subpopulations of cells during tumor treatment and response.	^372^
	Epigenetic states	KDM5A H3K4me3 H3K4me2	non-small-cell lung cancer	gefitinib erlotinib cisplatin	**Survival subpopulations of cells** in chemotherapy may shift the balance of cell populations toward resistance due to further epigenetic fixation.	^373^
		IRF CCL9 NF1 CD73 PD-L1	glioblastoma multiforme	PD-1 inhibitors	CSCs undergo stable transcriptional and epigenetic changes, leading to increased recruitment of **tumor-associated macrophages**.	^374^
Regulation of ferroptosis		SLC7A11 eIF2α ATF4 GSH ROS	triple-negative breast cancer	doxorubicin cis-platinum	The eIF2α/ATF4 axis up-regulates the expression of SLC7A11, promotes the synthesis of **GSH** and inhibits the accumulation of **ROS**.	^375^
		AXL MITF TGF-β GPX4	melanoma	BRAF inhibitors	Increased expression of AXL and decreased expression of MITF, can rely on the **lipid peroxidase pathway** to prevent ferroptosis.	^376^
Metabolism		Glucose FFAs CD36	breast cancer	antiangiogenic drugs	AAD limit the supply of glucose. Cancer cells use **alternative energy-producing mechanisms**, such as promoting lipolysis in fat cells, and activating the β-oxidation pathway to produce FFAs metabolism.	^377^
Tumor-derived exosomes	Induction of EMT	miR-155-5p GATA3 TP53INP1	gastric cancer	paclitaxel	Up-regulated **miR-155-5p** in drug-resistant cells can be delivered to sensitive cells via exosomes, inducing a malignant phenotype.	^378^
	Promotion of anti-apoptotic pathways	miR-32-5p PTEN	hepatocellular carcinoma	5-fluorouracil oxaliplatin gemcitabine sorafenib	Long-term exposure upregates **miR-32-5p**, activates the PI3K/Akt pathway, and further induces multi-drug resistance via exosomes.	^379^
	Drug effect	miR-365 CDA Triphospho -nucleotide	pancreatic ductal adenocarcinoma	gemcitabine	**miR-365** impaired activation of gemcitabine by upregulation of the triphospho-nucleotide pool and the induction of the enzyme cytidine deaminase.	^380^
	Signal transduction alteration	Heparinase ERK syndecan-1 proteoglycan	myeloma	bortezomib carfilzomib melphalan	Common exposure to used drugs enhanced exosome secretion and thus transport **heparinase** to unexposed cells, activating ERK signaling, and increasing syndecan-1 proteoglycan shedding.	^381^
ECM interactions	Single integrin	Integrinβ1 Src AKT	non-small-cell lung cancer	erlotinib gefitinib	**Integrin β1**/Src/AKT signaling pathway is a key mediator of acquired resistance to EGFR-targeted anticancer drugs.	^382^
	Multiple integrins	Integrinβ1 integrinβ4 ILK FAK	ovarian carcinoma	WX390	Matrix-attached carcinoma cells tolerate dual PI3K/mTOR inhibition by inducing an adaptive pro-survival response. **Integrin β1, integrin β4**, ILK, and FAK are engaged in this process.	^383^
Physical barriers		Compound of esters	breast cancer with brain metastases	capecitabine paclitaxel	Cancer cells colonize in **anatomical spaces** where drugs do not reach therapeutic concentrations.	^384^
ABC Transporters		MRP1	prostate cancer	calutamide flutamide	MRP1 catalyzes **the output of exogenous drugs**, which are often coupled to glutathione, glucuronic acid, or sulfate.	^385^

## The multi-stages of metastasis: A journey from origin to dissemination

In 1829, Recamier et al. first proposed the concept of “Metastasis” marking an early recognition of cancer metastasis.^15^ Over a century later, Bross et al. detected the cascade diffusion of metastases within the human body, further elucidating the process of cancer metastasis.^16^ Using ectopic organs, Hart et al. established the organ specificity of metastasis in 1980, laying the groundwork for a deeper understanding of cancer metastasis mechanisms.^17^ Advances in scientific technology have since uncovered numerous mechanisms underlying tumor metastasis, which have been translated into clinical practice. In 2000, Hanahan and Weinberg highlighted “Tissue invasion and metastasis” as hallmarks of cancer lethality,^18^ emphasizing the urgent need to elucidate the molecular mechanisms of cancer metastasis in 21st-century cancer research.

Genetic and phenotypic heterogeneity among cell populations within tumors play crucial roles in tumorigenesis and progression. In 1988, Steeg et al. identified the metastasis suppressor gene NM23 while screening melanoma cell lines with different metastatic potentials, linking it to lower tumor metastatic potential.^19^ Although many metastasis-related genes have been identified to date, the mechanisms of action for many of these genes remain incompletely understood. Chromosomal instability (CIN) is considered a hallmark of cancer, driven primarily by continuous errors during chromosome segregation in mitosis, contributing to tumor evolution. In 2018, Bakhoum et al. demonstrated the involvement of CIN in the regulation of the metastatic process by maintaining TCs’ autonomous response to cytoplasmic DNA,^20^ shedding new light on the intimate relationship between CIN and tumor metastasis and providing a novel perspective for deeper understanding of metastasis. Additionally, the phenomenon of cellular senescence in diploid human fibroblasts, first observed by Hayflick and colleagues, is considered a conservative response associated with various cellular stresses, including telomere shortening, carcinogenesis, and genetic toxicity.^21–23^ Senescence-induced genetic and metabolic changes are associated with tumorigenesis and cancer treatment response, holding significant implications for understanding tumor mechanisms.

Since the late 1860s, researchers have observed the existence of CTCs in the blood of cancer patients, present as both single cells^24^ and clustered structures.^25^ The observed higher metastatic potential of CTC clusters has established a robust theoretical framework for understanding the circulatory phase of tumor metastasis across various stages.^26^ Moreover, studies have demonstrated that CTCs capable of metastasizing to secondary organs can survive long-term in the bloodstream without undergoing apoptosis, highlighting the crucial role of cell death signal regulation in TC metastasis. Historically, cell death has long been considered a passive and unregulated process^27^ until the discovery of apoptosis executed by developmental pathways in the 1970s, the first example of programmed cell death.^28^ The capacity of CTCs to evade apoptosis during metastasis underscores the importance of researching anti-apoptotic mechanisms related to CTCs, which is vital for guiding strategies to prevent early metastasis. The interaction between platelets and cancer has attracted considerable attention since the late 1960s.^29^ Platelets can promote tumor growth, enhance immune evasion in the tumor circulation, and facilitate the long-term survival and successful metastasis of CTCs. In recent years, antiplatelet drugs like aspirin have attracted considerable attention for their potential role in this context.^30^ However, some CTCs that successfully reach secondary sites may temporarily remain in a static state without exhibiting proliferative phenotypes or triggering significant macroscopic metastasis.^31^ These cells, termed “dormant cancer cells” by Geoffrey Hadfield in 1954, remain quiescent within the affected tissues.^32^ Research on dormant cancer cells has continued to advance, and the development of advanced technologies enables better detection and definition of these rare cells, providing new opportunities for eradicating dormant cancer cells and preventing disease recurrence.^31^

**Fig. 2:**
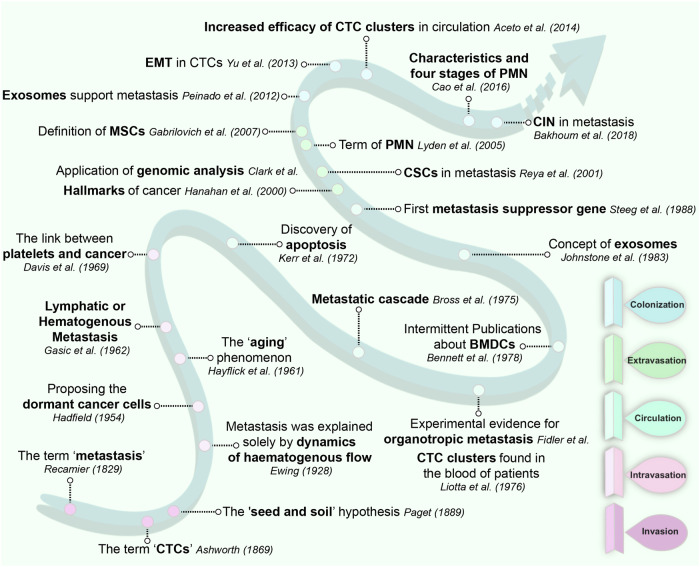
The research journey of multi-stage metastasis: a timeline perspective. Metastasis, the deadliest hallmark of cancer, stands as one of the pivotal questions of the 21st century, demanding precise elucidation of its molecular underpinnings. Researchers have made significant strides in elucidating fundamental concepts of multistage tumor metastasis, molecular markers, and cellular interactions driving metastatic dissemination. From early observations of tumor spread in the 19th century, such as Stephen Paget’s seminal “seed and soil” hypothesis, to today’s utilization of advanced imaging modalities and single-cell sequencing technologies, each milestone reflects our progressing understanding of the metastatic process. Clarification of key signaling pathways such as EMT, angiogenesis, and immune evasion mechanisms has provided crucial insights into how cancer cells acquire migratory and invasive capabilities. Ongoing research efforts, including investigations into the role of the tumor microenvironment, EV-mediated intercellular communication, and the impact of genetic heterogeneity on multistage metastasis, will continue to unveil new aspects of organ-specific metastasis. In this figure, we focus on key discoveries and milestones in cancer metastasis research, illustrating the timeline of research history and highlighting trajectories of discovery

In tumor metastasis research, organ-specific phenomena are referred to as organ tropism, a persistent enigma in cancer research. The occurrence of organ tropism is contingent upon the formation of PMNs at secondary sites. The concept of PMNs can be traced back to Stephen Paget’s “seed and soil” hypothesis in 1889,^33^ further consolidated by Isaiah Fidler’s experimental evidence in 1976.^34^ The term PMNs was first introduced by Lyden in 2005,^35^ and subsequently, in 2018, Cao et al. detailed the characteristics of PMNs and outlined four stages of metastasis: priming, licensing, initiation, and progression, thereby refining the theoretical framework.^36^ In 1983, Rose Johnstone et al. first named the EVs secreted in culture media as exosomes.^37^ In recent years, as research on PMNs has deepened, it has been recognized that exosomes, as key participants in cell-to-cell communication, play crucial roles in the formation of PMNs. With advances in the study of exosomes and continuous technological progress, exosomes have now become one of the highly regarded fields in biomedical research.^12^ (Fig. 2).

## Genesis of invasion: Progression dynamics at the primary site

### Invasion

#### Appearance of genetic intratumoral heterogeneity

Genomic analysis reveals that abnormal mutations in tumor suppressor genes such as TP53, RB1, BRCA1, and cancer-related genes like C-MYC, KRAS, epidermal growth factor receptor (EGFR), and ALK in normal cells can lead to unchecked cell growth and proliferation, which are closely linked to the onset and progression of various cancer types. Research indicates that these driver genes also regulate TC migration and invasion, contributing to metastatic tendencies. For instance, in lung metastatic lesions of esophageal squamous cell carcinoma (SCC), significant upregulation of colony-stimulating factor-1 (Csf-1) is observed in a p53-R172H-dependent manner, which, through its receptor Csf-1r and coordination with Stat3 phosphorylation and EMT, enhances TC invasion and lung metastasis.^38^ In vivo experiments have demonstrated that AFAP1-AS1 interacts with Smad nuclear-interacting protein 1 to inhibit ubiquitination and degradation of c-Myc protein, thereby promoting lung cancer cell migration and invasion.^39^ Additionally, upregulation of c-Myc can further facilitate lung cancer metastasis by promoting the expression of ZEB1, ZEB2, and SNAIL genes. Moreover, the elevated RAS-MAPK signaling pathway promotes angiogenesis and TGF-β signaling through aberrant crosstalk between cancer stem cells (CSCs) and their microenvironment, leading to the activation of downstream phosphoinositide 3-kinase (PI3K)-AKT-mTOR signaling and regulating the progression of benign papillomas to invasive malignant tumors.^40^ Independent studies have determined that cancer driver genes may be reconnected to activate cell death, suggesting that the coexistence of driver gene mutations and cell death pathways may be feasible within the organism.^41^ Researchers have developed a new class of molecules called TCIPs (transcription/epigenetic chemistry proximity inducers), which recruit endogenous cancer drivers or downstream transcription factors to the promoters of cell death genes, inhibiting their expression from both transcriptional and epigenetic perspectives.^42^ Despite these advances, the potential synergistic mechanisms between these gene drivers in promoting tumor development and metastasis remain unclear. For example, in pancreatic ductal adenocarcinoma (PDAC), co-occurrence of KRAS mutations with TP53 gene alterations is observed in 70% of patients. This suggests that a deeper understanding of the complex interactions between oncogenes and mutated tumor suppressor genes may unveil new therapeutic approaches to mitigate metastasis by reversing cooperative mechanisms.^43^

While PDAC can be classified into multiple subtypes based on gene expression profiles, which may be associated with prognosis and treatment response,^44^ the mutations and expression levels of driver genes in PDAC progression appear to be conservative. Specific gene mutations directly implicated in cancer metastasis dissemination have not been distinctly identified.^45^ However, an analysis of RNA splicing data from a large cohort of primary and metastatic PDAC, the study reveals that alternative splicing events play a significant role in PDAC progression. Splicing events regulated by myosin phosphatase RHO-interacting protein and RBFOX2 are associated with PDA metastasis, cytoskeletal remodeling, and focal adhesion formation induction.^46^ Similarly, in breast cancer (BC) metastasis, the splicing factor SNRPA1 interacts with hundreds of structure-enhancing splicing enhancers enriched near cassette exons to promote cassette exon inclusion, enhancing BC cell invasion and lung colonization.^47^

Genetic alterations occurring at the gene level exhibit DNA sequence disruption and irreversibility, whereas reversible epigenetic changes modulate gene expression programs promoting tumor initiation, characteristic phenotypes, and functionality, thus advancing drug development. Major epigenetic modifications include DNA methylation and histone mark patterns.^48^ Studies reveal that Type I interferons (IFNs-I) trigger epigenetic regulator demethylase 1B (KDM1B) during immunogenic chemotherapy, promoting reversible transcriptional rewiring, facilitating TC adaptability, stemness establishment, immune escape, and enhancing tumor invasiveness.^49^ CSCs have been implicated in tumor progression, drug resistance, and metastatic tumor formation.^50^ For instance, progressive cholangiocarcinoma exhibits overexpression of peroxisome proliferator-activated receptor γ coactivator-1α (PGC-1α), while Fat1 loss in skin SCC favors CSC maintenance and invasiveness.^51–53^ Histone variants and their chaperones have emerged as key epigenetic regulators, making chromatin highly responsive to environmental signals. Pathways inducing metastasis modulate histone chaperones to reduce canonical histone incorporation into chromatin, promoting H3.3 variant deposition at the promoters of poor prognosis genes and metastasis-inducing transcription factors, crucially regulating tumor invasive features.^54^ While H3 mutations are necessary for certain tumor initiation and progression, they are not sufficient alone, often requiring concurrent alterations in driver genes, which may offer more precise therapeutic targets than H3 mutations alone.^55^ Moreover, in melanoma models, embryonic stem cell factor SALL4 negatively regulates invasiveness by interacting with histone deacetylase (HDAC) 2 and directly binding to a set of invasive genes, implicating HDAC2 in SALL4-dependent regulation of melanoma phenotype switching.^56^

CIN, characterized by persistent errors in chromosome segregation during mitosis, is a hallmark of cancer. This instability promotes metastasis by maintaining autonomous TC responses to cytoplasmic DNA and acts as a primary driver of tumor evolution.^20,57^ In the invasive progression of non-small cell lung cancer (NSCLC), the activity of APOBEC3B induces incomplete replication and replication stress in the genome, triggering erroneous chromosome segregation, tightly associated with CIN and somatic mutation heterogeneity. These processes exacerbate tumor invasiveness, thus impacting the biological characteristics of tumors.^58^ Recent studies have also identified protein mutations that regulate higher-order chromatin structures in certain cancers, which are closely linked to increased tumor invasiveness.^59^ For instance, in triple-negative breast cancer (TNBC), topologically associated domain boundaries downstream of the MMP8 gene isolate MMP genes into two inversely correlated expression clusters, closely correlated with TNBC invasiveness enhancement and poor patient prognosis.^60^ Further research reveals significant disruption in the spatial partitioning of open and closed compartments in the tumor genome, identifying recombination segments between classical A and B compartments. These alterations in topological structures not only impact gene expression patterns but also likely regulate tumor invasion and metastasis programs, contributing to malignant tumor progression.^61^ In summary, chromosomal-level heterogeneity significantly influences tumor invasiveness, and in-depth research into these heterogeneities not only aids in understanding the mechanisms of cancer initiation and progression but also provides new avenues and directions for future therapeutic strategies.

#### Subversion of the tumor immune microenvironment

For primary TCs to develop effectively, they must evade recognition and destruction by the immune system.^2^ This process involves the reprogramming of adaptive and innate immune cells into distinct subpopulations or inducing functional instability and suppressive metabolic conditions.^62^ Chronic inflammation associated with cancer, for example, leads to the expression of pro-inflammatory cytokines, which drive the differentiation of bone marrow (BM) cells into myeloid-derived suppressor cells (MDSCs) such as macrophages, granulocytes, neutrophils, and dendritic cells. These cells accumulate in the circulation of cancer patients and are recruited by growth factors released by cancer cells, which have the capacity to inhibit the proliferation and activity of cytotoxic T lymphocytes (CTLs), promote angiogenesis, and enhance the survival of TCs.^63^ Simultaneously, the immunogenicity of neoplastic lesions is also altered by evolutionary constraints the host immune response imposes.^64^ These co-evolutionary interplays involving TCs and the immune system, termed “immunoediting”, encompass three phases: elimination, equilibrium and escape.^65^

The immune microenvironment consists of a diverse array of cells, including macrophages, natural killer (NK) cells, neutrophils, B cells, and T cells. Experimental observations have shown that the presence or absence of these cells varies across different diseases and stages of progression.^66^ For example, macrophages, regulating TC spread, dormancy, and stem cell activity, present M1-like pro-inflammatory phenotype during the initial stages of tumorigenesis,^67^ while a significant polarized macrophage infiltration can be observed in most developed solid tumors.^68^ Both NK cells and neutrophils, similarly pivotal in innate immunity, undergo the transformation driven by the elevated concentration of TGF-β within the TME. NK cells shift into intermediate type 1 innate lymphoid cells without cytotoxicity, whereas neutrophils polarize towards a pro-tumor direction.^69,70^ In addition, the anomalous differentiation process fueling tumor progression is also evident in adaptive immune cells. Recent research has revealed that Foxp3+ regulatory T cell (Treg) aggregation and B cell clonal expansions and Ig subclass switch events, contributing to the establishment of immunosuppressive TME, have been unveiled in many malignancies.^71,72^ For example, in a BC lung metastasis paradigm, CD4+ T cells can be converted into FOXP3+ Treg cells in a TGF-β-dependent manner, a process facilitated by regulatory B cells (Bregs), thereby promoting tumor progression.^2^ Furthermore, tumors can induce the differentiation of circulating B-cell precursors into metastasis-promoting Bregs and macrophage-like B cells through the secretion of thymic stromal lymphopoietin and macrophage colony-stimulating factors (M-CSF) respectively. This cascade further results in the contraction of the CD4+ T cell pool and the generation of FOXP3+ Treg cells, amplifying cancer progression and metastasis.^73,74^

**Fig. 3:**
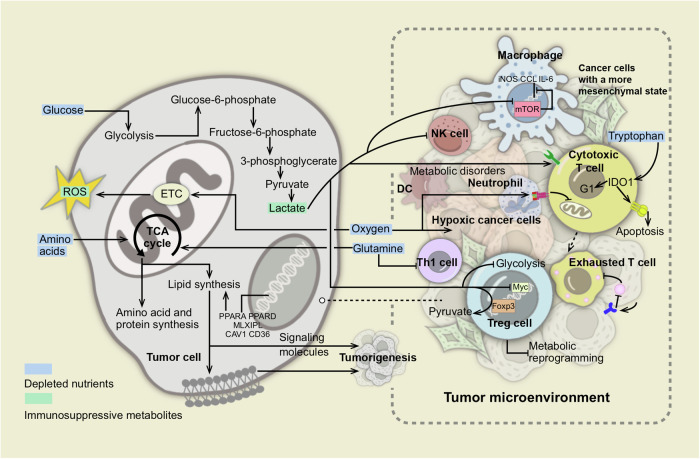
The metabolic interventions with immune cells exited in TME. Due to the limited availability of oxygen, nutrients, and other substances in the TME, it presents a challenging milieu where cancer cells must adapt to survive under harsh conditions. In response, these cells undergo metabolic alterations involving three main nutrients: carbohydrates, amino acids, and lipids. For instance, genes involved in cellular fatty acid uptake (CAV1, CD36) and de novo synthesis (PPARA, PPARD, MLXIPL) are frequently amplified specifically in metastatic tumors. Lipids synthesized de novo can modulate membrane fluidity, impacting interactions between tumor cells and immune cells, thereby exerting anti-tumor phagocytic functions. Additionally, these lipids can act as signaling molecules, triggering oncogenic cascades. Deprivation of Gln in the TME is known to impair differentiation of Th1 cells, a subset of T helper cells crucial for coordinating anti-tumor immune responses. Moreover, enzymes such as indoleamine 2,3-dioxygenase 1 (IDO1) catalyze tryptophan oxidation, inducing T cells into the G1 phase of the cell cycle and fostering Fas-mediated cell apoptosis. In the prevalent hypoxic conditions of solid tumors, oxygen deprivation coordinates TCR stimulation and mitochondrial dysfunction in T cells, resulting in a state of exhausted T cells that suppress anti-tumor immunity. Furthermore, acidic pH levels in the TME exacerbate immune suppression by altering the metabolic pathways of immune cells. Low pH induces metabolic dysregulation in T cells, activating checkpoint molecules and promoting immune suppression. Additionally, low pH inhibits mTOR and NK cell anti-tumor activity, suppressing expression of iNOS, CCL2, and IL-6 in M1-type macrophages. In Treg, the transcription factor Foxp3 is conducive to the oxidation of L-lactic acid to pyruvate. Meanwhile, the accumulated lactic acid can enhance oxidative phosphorylation and the oxidation of nicotinamide adenine dinucleotide by inhibiting Myc and glycolysis, thus participating in the metabolic reprogramming process of Treg cells. Furthermore, activation of Toll-like receptor (TLR) signaling in tumor cells disrupts cAMP production, enhancing anti-tumor immune responses. Understanding the metabolic complexity within the TME is crucial for developing effective therapeutic approaches. Targeting these aberrant metabolic reprogramming processes holds promise for enhancing current immunotherapies and improving outcomes for cancer patients

Effector tumor-infiltrating lymphocytes (TILs) require a high metabolic rate to perform their functions, necessitating significant energy resources. Consequently, the oxygen and nutrients consumed by TCs, along with the production of metabolic waste, may obstruct the vital metabolic pathways and functional state of TILs.^75^ (Fig. 3) For example, under hypoxic conditions, the combined effect of T cell receptor (TCR) stimulation and exposure to TC-conditioned medium can impair mitochondrial function in TILs, leading to epigenetic reprogramming associated with the onset of exhaustion.^76^ Furthermore, hypoxia may influence the capacity of TCs to transition between epithelial and mesenchymal phenotypes. In the TME dominated by mesenchymal TCs, the cytotoxicity of CTL and NK cells is markedly diminished, thereby fostering the development of an immunosuppressive state.^77,78^ TCs prominently rely on glycolysis to metabolize glucose and generate lactic acid, even under normoxic conditions.^79^ In an environment characterized by low glucose and abundant lactic acid resulting from this process, Treg and M2 macrophages exhibit distinct metabolic advantages compared with T cells, NK cells, and M1 macrophages possessing anti-tumor activity.^80,81^ Additionally, the reduction in antitumor immunity can also be brought on by shortages in non-essential amino acids including glutamine, arginine, and asparagine in TME as well as essential amino acids like tryptophan and methionine.^82,83^ However, recent studies have revealed that glucose is not strictly restricted in TME and is preferentially allocated to TILs.^84^ Concurrently, comparisons of nutrient levels between tumor interstitial fluid and plasma indicate that not all nutrients are depleted in the TME.^85^ This illustrates that immunosuppression in glycolytic tumors does not stem directly from inadequate nutrition; instead, it arises from extensive alterations within the TME that disrupt cellular innate programming, is determined by a variety of factors including tumor intrinsic factors, anatomical location, systemic metabolic changes, and tissue origin, etc.^84,85^

**Fig. 4:**
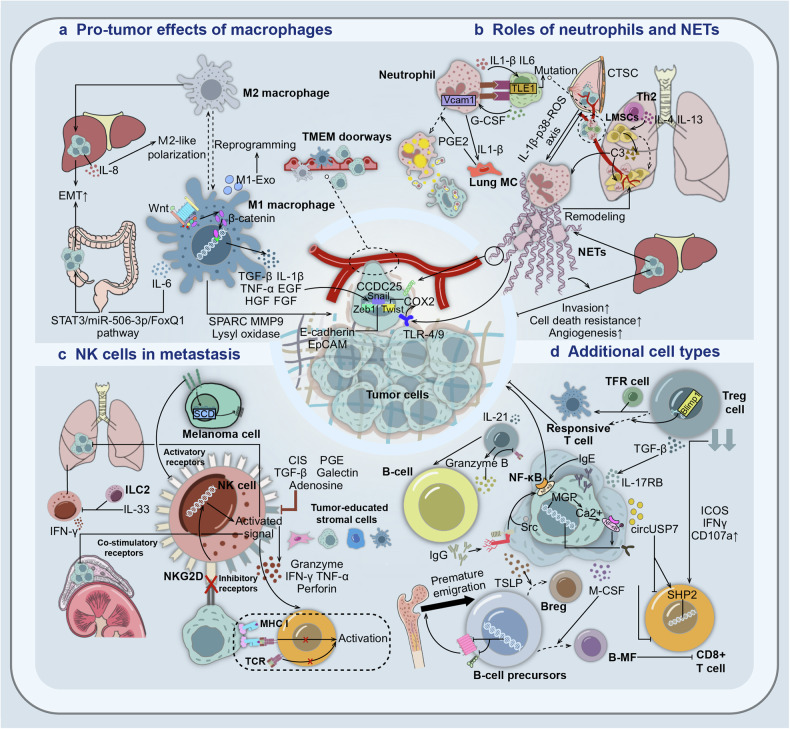
Pro-tumor functions exerted by various immune cells during tumor progression. **a** The remodeling process of the tumor ECM is regulated by macrophages. WNT/β-catenin signaling regulates the release of inflammatory factors. Meanwhile, IL-6 can induce EMT to enhance CRC migration and invasion. In HCC, IL-8 stimulates M2-type polarization of TAMs, promoting EMT. The migrating tumor cells were preferentially located near the tumor microenvironment of metastasis (TMEM) gate after escaping from the tumor cell nest. **b** Neutrophils transport lipids into tumor cells through the macropinocytosis lysosome pathway. Th2 cell-derived IL-4/IL-13 promotes the formation of NETs to reshape PM niches. Cathepsin C (CTSC) promotes the formation of NETs. HCC-induced NETs activate TLR4/9 while inducing an inflammatory response by up-regulating COX2. Additionally, NETs can bind to CCDC25 on cancer cells as a chemokine. IL1β and IL6, as well as Vcam1 gene transcripts, play an important role in the formation of CTC-neutrophil clusters. TLE1 mutations in CTCs increase G-CSF and form a positive feedback loop with other cytokines. **c** NK cells exist to counteract the mechanism by which tumor cells down-regulate the expression of MHC I. The function of NK cells is regulated by both active and inhibitory receptors. Silencing of NKG2DL can lead to the failure of NK cells to activate, inducing potential immune evasion in SCLC and neuroblastoma. Tumor-derived molecules, tumor-associated stromal cells, and tumor cells exert inhibitory effects on NK cells. IFN-γ production and the overall amount of IFN-γ positive NK cells in the lungs were substantially reduced in mice treated with IL-33 which could diminish NK cells’ capacity when combined with type 2 innate lymphoid cells (ILC2). **d** TGF-β induces cancer cells to produce IL-17RB. Knockout of Blimp1 in Treg reprograms it into responsive T cells, promoting IgE deposition and secondary macrophage activation process. Clearance of Tregs restores the function of CD8+ T cells based on the significantly increased expression of ICOS, IFNγ and CD107a. CircUSP7 can inhibit the secretion function of CD8+ T cells or the expression of Src homology region 2-containing protein tyrosine phosphatase 2 (SH2P2). Matrix Gla protein (MGP) enriches intracellular free calcium and promotes CD8+ T cell depletion. IgG activates the NF-κB pathway and promotes tumor metastasis. Down-regulation of CXCR4 and VLA4 leads to premature emigration from BM. Thymic stromal lymphopoietin (TSLP) induces B-cell precursors to differentiate into Breg, while M-CSF promotes their differentiation into macrophage-like cells (B-MF). IL21-secreting Tregs stimulate B cell activation, and the granzyme B produced can degrade part of TCR

The buildup of an immunosuppressive TME is a key factor in tumor development and treatment tolerance. Recent research has focused on elucidating the molecular mechanisms driving the body’s immunosuppressive state and the functional instabilities of immune cells at primary sites during tumor progression. In TCs, the upregulation of transcription factors that regulate intercellular junction structure, along with the downregulation of E-cadherin and epithelial cell adhesion molecules, is driven by inflammatory factors secreted by tumor-associated macrophages (TAMs). This process facilitates cancer cell progression.^86^ And in another experience, IL-6, derived from TAMs, was found to enhance the invasion capacity of colorectal cancer (CRC) cells through the induction of EMT via the STAT3/miR-506-3p/FoxQ1 pathway.^87^(Fig. 4a) In the lung mesenchyme, the IL1β-pulmonary MC-PGE2-neutrophil EP2 axis can initiate intra-neutrophil lipid storage processes and translocation into TCs via the micropinocytosis-lysosome pathway during neutrophil infiltration, which results in higher proliferation and greater pro-survival capacity of TCs under nutrient deprivation conditions.^88^ Clinical observations that increased infiltration of AGR2+ tumor-associated neutrophils correlates with poor prognosis in CRC patients support this irregular function of neutrophils.^89^ (Fig. 4b) Direct cytotoxic effects mediated by perforin and the secretion of inflammatory cytokines can be stimulated by ligands expressed on the surface of cancer cells, such as NKG2DL.^90^ Nevertheless, in melanoma cells, overexpression of the nerve growth factor receptor NGFR (CD271 / p75NTR) will downregulate NK cell activation ligands while upregulating fatty acid stearoyl coenzyme A dehydrogenase, which reduces NK cell infiltration, cell degranulation and the sensitivity of melanoma cells to NK cell-mediated tumor killing.^91^ (Fig. 4c) It is well-established that activated T lymphocytes can effectively destroy TCs upon recognizing peptides presented by the class I major histocompatibility complex (MHC-I). However, mutations in MHC-I or loss of heterozygosity on the malignant cell surface can impair antigen presentation.^92^ For example, in PDAC, MHC-I is enriched in autophagosomes and lysosomes and reduces its own expression through the NBR1-mediated autophagy-lysosome pathway.^93^ Similarly in small-cell lung cancer, the polycomb repressive complex 2 can act as a transcriptional repressor to silence the MHC-I class I antigen processing pathway at the molecular level, which leads to the suppression of CD8+ T cells and the generation of cytokines.^94^ Furthermore, tumor-secreted factors exert an indispensable role in molding the phenotype of T cell functional dysregulation. In NSCLC, tumor-derived circUSP7 is synthesized and released in an exosomal manner, hindering the secretion of IFN-γ, TNF-α, granzyme-B, and perforin by CD8+ T lymphocytes or upregulating Src homologous region 2-containing protein tyrosine phosphatase 2 expression to inhibit CD8+ T cell function.^95^ Additionally, matrix Gla protein, a calcium-binding matrix protein secreted by CRC cells and significantly upregulated, can enhance intracellular calcium ion levels, promote NF-κB phosphorylation, activate PD-L1 expression, and contribute to CD8+ T cell depletion.^96^ (Fig. 4d).

#### The systemic macroenvironment modulation

**Fig. 5:**
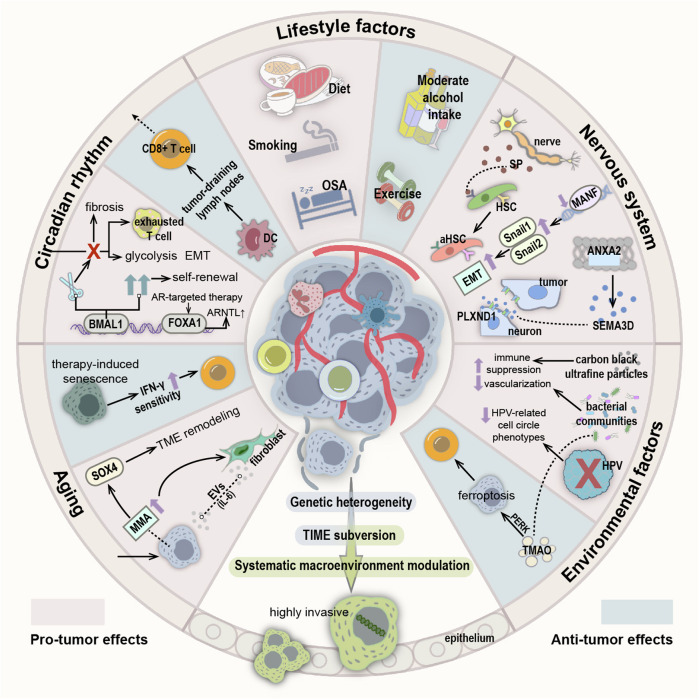
Influences on tumor invasiveness from lifestyle, neurological, environmental, aging, and circadian perspectives. The invasiveness of tumors is influenced by multiple factors, including genetic heterogeneity, tumor immune microenvironment subversion, and systemic macroenvironmental modulation. In addition to recognized dietary risk factors, lifestyle factors such as smoking, alcohol consumption, exercise, and sleep play significant roles in the proliferation, invasion, and progression of tumors. For instance, peripheral neurons within the TME can secrete neuropeptides that activate normal aHSCs, thereby promoting invasion and metastasis of HCC. Specific MANF deficiency in the liver upregulates Snail1 and Snail2 levels, thereby promoting EMT and accelerating HCC progression. In the context of PDAC, the invasion of DRG cells depends on the expression of ANXA2 and axon guidance molecule SEMA3D. Functional modulation of ANXA2 influences SEMA3D secretion and enhances tumor cell migration and invasion by binding to PLXND1 receptors on DRG surfaces. Furthermore, long-term exposure to incomplete combustion products such as ultrafine particles of carbon black in air increases glycolysis and lactate production, resulting in an immunosuppressive microenvironment. Experimental observations reveal that bacterial communities predominantly inhabit microecological niches with lower vascularization and higher immunosuppression. Additionally, cell populations lacking HPV expression exhibit reduced HPV-related cell cycle phenotypes, weaker treatment responses, and enhanced invasive capabilities. Bacterial metabolite TMAO activates the PERK pathway to induce ferroptosis in tumor cells, thereby enhancing CD8+ T cell-mediated anti-tumor immune responses. With advancing age, invasive cancer cells produce increased levels of MMA, inducing SOX4-related remodeling of the TME, activating fibroblasts, and reciprocal secretion of IL-6-carrying EVs involved in cancer progression. In human tumor cells undergoing therapy-induced senescence, upregulation of IFN-γ receptors triggers CD8+ T cell-mediated tumor rejection, enhancing the efficacy of immunotherapy. Disruption of circadian rhythms plays a crucial role in T cell exhaustion, with malignant cells exhibiting enhanced glycolysis and EMT activation linked to high circadian disruption scores. Deleting key clock transcription factor BMAL1 exacerbates fibrotic phenotypes across various tumors. Finally, in glioblastoma stem cells, strict regulation of self-renewal by the BMAL1 gene supports optimal cell growth. Resistance to AR-targeted therapy in PCa cells correlates with extensive reprogramming of FOXA1 loci and enrichment of clock component ARNTL. Rhythmic transport of DC to tumor-draining lymph nodes controls CD80-dependent circadian responses of specific CD8+ T cells, thereby enhancing therapeutic outcomes

Diet is a well-established risk factor for various cancers. Research on various nutrients or plant and chemical substances reveals associations between dietary factors and cancer risk. For instance, research using animal models of CRC has shown that a ketogenic diet, which restricts carbohydrates but includes sufficient dietary fiber, provides optimal protection against intestinal tumor development. The ketone body β-hydroxybutyrate (BHB) can replicate the tumor-suppressing effect of the ketogenic diet through surface receptor Hcar2 and transcriptional regulator Hopx, suggesting that the tumor-inhibitory effect of diet can be replicated by supplementing metabolites. Thus, the BHB-mediated pathway acting in concert with other therapeutic modalities will potentially become an exemplar of “metabolic therapy.“ ^97^ Moreover, research indicates that a high-fat diet (HFD) increases TC uptake of fat, alters fatty acid distribution in the tumor microenvironment (TME), and impairs the infiltration and function of CD8+ T cells, thereby promoting tumor growth.^98^ In leukemia patients, inhibition of fat mass and obesity-associated protein sensitizes leukemia cells to cytotoxicity from T cells, overcoming immune evasion induced by hypomethylating agents, and inhibiting tumor progression.^99^ Obesity, often associated with an HFD, is also a known risk factor for cancer and is linked to poor prognosis in various malignancies. In BC, the upregulation of ACSBG1 and SLC6A8 in the obese microenvironment of cancer cells supports the production of phosphocreatine by promoting ATP generation and the uptake of creatine from adipocytes, ensuring continued synthesis of metabolic processes even during hypoxia, thus promoting tumor progression.^100^ In recent years, the consumption of artificial sweeteners as zero-calorie sugar substitutes has significantly increased. However, studies have found that high doses of sucralose in mice affect T cell membrane organization, reduce TCR signal transduction and intracellular calcium mobilization efficiency, limiting T cell proliferation and differentiation, and demonstrating reduced antigen-specific responses of CD8+ T cells in subcutaneous cancer models, thereby promoting tumor progression.^101^ Beyond the direct effects of specific nutrients and chemicals, the influence of dietary factors on cancer risk is also contingent on cancer type, other risk factors such as age, lifestyle factors, comorbidities, and the composition of the gut microbiota.^102^ (Fig. 5).

Aging induces stable cell cycle arrest and the secretion of various factors that can remodel tissue environments, exerting both positive and negative effects on the organism, particularly in the context of cancer.^103^ For example, with increasing age, invasive cancer cells gradually producing methylmalonic (MMA) acid, inducing SOX4, which is associated with remodeling of the TME,^104^ and activate fibroblasts. This activation leads to mutual secretion of EVs loaded with IL-6 between TCs and fibroblasts, which participate in regulating cancer progression, drug resistance, and metastasis.^105^ In certain circumstances, these secretion programs can also stimulate the immune clearance of senescent cells. In human TCs undergoing therapy-induced senescence, an upregulation of IFN-γ receptors is observed compared to proliferating cells, rendering senescent cells highly sensitive to the microenvironmental IFN-γ, triggering CD8+ T cell-mediated tumor rejection in an immunoreactive liver cancer model, contributing to the anti-tumor activity of immunotherapy.^104^

Additionally, lifestyle factors such as smoking, alcohol consumption, exercise, and sleep play crucial roles in the proliferation, invasion, and progression of tumors. Smoking constitutes a primary risk factor for lung cancer, with cigarette smoke extract (CSE) and benzo[a]pyrene enhancing osteopontin expression levels. This upregulation facilitates the recruitment and adhesion of mesenchymal stem cells to lung cancer cells via JAK2/STAT3 signaling and promotes the formation of tumor-associated mesenchymal stem cells through osteopontin receptors (integrins αvβ1, αvβ3, αvβ5, or CD44), thereby enhancing lung cancer cell migration and invasion.^105^ Moreover, in NSCLC, M2-TAM induced by EVs containing circEML4 from CSE can promote tumor progression through ALKBH5-mediated m6A modification of SOCS2. CircEML4 from TAM-derived EVs also serves as a diagnostic biomarker for NSCLC.^106^ Experimental data indicate that cigarette-derived 4-(methylnitrosamino)-1-(3-pyridyl)-1-butanone activates the TMUB1/AKT pathway via METTL14/YTHDF2-mediated m6A modification, significantly correlating with increased cancer invasion and metastasis risk.^107^ The relationship between alcohol consumption and cancer remains inconclusive in both epidemiological studies and animal models. However, in a mouse model of BC metastasis, researchers found that long-term moderate alcohol intake (ranging from 0.5% w/v to 2.0% w/v) downregulates oncogenes associated with primary tumors and modulates the immune and metabolic systems in metastatic cancer to inhibit tumor progression.^108^ Exercise, known to prevent cancer occurrence and recurrence, is often associated with creatine supplementation to increase muscle mass and enhance athletic performance. However, dietary intake or de novo synthesis of creatine mediated by GATM may upregulate Snail and Slug expression through Smad2 and Smad3 phosphorylation activated by monopolar spindle 1, thereby driving tumor invasion and metastasis. Hence, caution is advised when considering dietary creatine for improving muscle mass or treating diseases.^109,110^ Obstructive sleep apnea is associated with increased incidence and mortality of lung cancer. Chronic intermittent hypoxia, which triggers the stability of HIF-1α/ATAD2, may determine lung cancer invasiveness through interactions with mitochondrial reactive oxygen species (ROS) and cancer cell stemness.^111^ Furthermore, based on cell-based experimental models and mouse models, melanoma cells are susceptible to intermittent hypoxia akin to that induced by sleep apnea. Fragmented sleep and other immune or metabolic changes arising from excessive sleep-disordered breathing also play significant roles.^112–114^ In CRC, chronic sleep deprivation promotes miR-223-3p expression in colon cancer cells via GABA, leading to downregulation of E3 ubiquitin ligase CBLB and inhibition of cMYC ubiquitination. Concurrently, extracellular miR-223-3p promotes M2-like macrophage polarization, resulting in IL-17 secretion and further enhancing the proliferation and invasion of colon cancer cells.^115^

The circadian rhythm governs temporal physiological regulation to maintain internal balance, playing a critical role in tumorigenesis and facilitating the establishment of cancer hallmarks.^116,117^ Analysis across various cancer types reveals that circadian rhythm disruption plays a pivotal role in T cell exhaustion, while malignant cells with high circadian rhythm disruption scores exhibit glycolysis and EMT activation states associated with poor prognosis.^118^ Dysregulation of circadian rhythm genes synergistically enhances intratumoral heterogeneity, further promoting adverse outcomes. Studies show that disrupting the circadian rhythm, such as through the deletion of the brain and muscle ARNT-like 1 (Bmal1) gene—encoding a key clock transcription factor—worsens fibrotic phenotypes in various tumors, thereby accelerating tumor growth and enhancing metastatic potential.^119^ In glioblastoma stem cells, strict dependence on core clock transcription factors BMAL1 and CLOCK is observed to promote self-renewal and metabolism for optimal cell growth.^120^ Moreover, both innate and adaptive immune responses, which exert immune surveillance function, exhibit circadian rhythms, regulating the host’s anti-tumor immune response and treatment response. For instance, the rhythmic transport of DCs to tumor-draining lymph nodes controls the CD80-dependent circadian response of specific CD8+ T cells, thus synchronous immune therapy with DC function yields better efficacy.^121^ However, in prostate cancer (PCa), resistance to androgen receptor (AR)-targeted therapy is also linked to circadian rhythm. Epigenomic analysis reveals that treatment-induced FOXA1 sites enriched with clock components ARNTL in PCa cells experience massive reprogramming towards active cis-regulatory elements determining pro-survival signals. Knocking out ARNTL significantly reduces PCa cell growth.^122^

Increasing evidence suggests that the nervous system plays a central role in the pathogenesis of cancer, with neuro-cancer crosstalk emerging as a key regulatory factor in cancer initiation and progression.^123^ The peripheral nervous system is an integral component of the TME, with tumors recruiting peripheral nerves into the TME to promote tumor growth through various mechanisms.^124^ Studies have found that peripheral neurons in the TME can secrete neuropeptides such as substance P (SP), which act on the SP/NK-1R signaling pathway, activating normal hepatic stellate cells (HSCs) to become activated HSCs (aHSCs), thereby promoting invasion and metastasis of hepatocellular carcinoma (HCC).^125^ Supportive cells also play crucial roles in the nervous system. Research has shown that cancer-activated Schwann cells (SCs) collectively contribute to the tumor-activated Schwann cell trajectory. Dynamic SCs form tracks, acting as cancer pathways and exerting forces on cancer cells to enhance their motility, thereby facilitating cancer cell migration and invasion.^126^ In HCC, levels of midbrain astrocyte-derived neurotrophic factor (MANF) mRNA and protein are lower compared to adjacent non-cancerous tissues. Liver-specific deletion of MANF leads to upregulation of Snail1 + 2 and promotes EMT, accelerating HCC progression.^127^ Perineural invasion is a distinct pathological feature of PDAC associated with poor prognosis. Studies have found that PDAC TC invasion of dorsal root ganglion (DRG) cells depends on the expression of membrane-associated protein A2 (ANXA2) and axon guidance molecule SEMA3D. Functional ANXA2 regulates SEMA3D secretion, binding and activating the receptor PLXND1 on DRG to increase TC migration and invasion activity.^128^ Similarly, in cancers of unknown primary (CUP), a novel activating mutation in the axon guidance gene PLXNB2 can maintain proliferative autonomy in an EGFR-dependent manner and confer invasive properties to CSCs isolated from CUP, promoting tumor progression.^129^ Thus, the identification of axon guidance molecules and axon guidance genes may provide guidance for the development of novel genetic biomarkers for tumor management.

Environmental factors, both endogenous and exogenous, can alter the metabolism, growth patterns, and functions of TCs, shaping the TME and participating in regulating tumor progression.^130^ Long-term exposure to carbon black ultrafine particles generated from incomplete combustion of organic compounds in air increases PD-L1 + PD-L2 + CD206+ antigen-presenting cells, exhausted T cells, and Treg cells. Lung macrophages containing these ultrafine particles exhibit selective mitochondrial structural damage, leading to reduced aerobic respiration and increased glycolysis and lactate production., This shift creates an immunosuppressive microenvironment, promoting tumor incidence and enhancing early metastasis by increasing tumor invasiveness.^131^ Through in situ spatial analysis and single-cell RNA sequencing techniques, it is found that in oral SCC and CRC, the distribution of bacterial communities is not random. They are primarily located in microecological niches with lower vascularization, higher immune suppression, and epithelial cell functions promoting cancer progression compared to bacteria-negative tumor regions.^132^ Moreover, there is considerable cellular diversity within and between tumors in HPV-related and HBV-related cancers. For example, a subset of cells lacking or suppressing HPV expression exhibits reduced HPV-related cell cycle phenotypes, diminished treatment response, and increased invasion, correlating with poor prognosis.^133,134^ Therefore, the diversity of viral expression must be considered in the diagnosis and treatment of virus-related tumors, which significantly impacts prognosis. In triple-negative TNBC, patients with higher levels of trimethylamine N-oxide (TMAO), a bacterial-related metabolite, in plasma show better responses to immunotherapy. This is attributed to TMAO’s ability to trigger TC ferroptosis through activation of the endoplasmic reticulum stress kinase PERK, thereby augmenting the in vivo anti-tumor immune response mediated by CD8+ T cells.^135^ These findings underscore the potential of microbial metabolites in enhancing treatment effectiveness by modulating the TME, presenting a promising avenue for novel therapeutic interventions.

### Intravasation

Circulation constitutes a pivotal stage in the distant metastasis of TCs. Throughout tumor progression, within the primary sites, genetically heterogeneous TCs undergo selective clonal expansion and components of the TME endure the reprogramming toward a pro-tumorigenic phenotype, both of which afford certain TCs the capability to infiltrate the circulatory system and disseminate to distant organs.^57^ In patients with late-stage HCC, levels of sEV-vWF and sEV-CLTA are elevated compared to normal levels. These, by modulating downstream factors such as VEGF-A, fibroblast growth factor 2, and basigin, reshape the microvascular niche, thereby enhancing HCC cancerous properties, interrupting endothelial integrity, and inducing angiogenesis.^136,137^ Additionally, a potential reliance on epigenetic modifications may hold greater significance than previously acknowledged in tumor metastasis. Within tumor clusters, the epigenetic activation of key Adherent-to-Suspension Transition factors induces a phenotypic switch, achieving the capacity to detach from the primary tumor and survive in the bloodstream through the induction of anoikis resistance via hemoglobin genes and the global suppression of integrin and ECM components via inhibiting the YAP-TEAD axis.^138^

## Navigating the circulatory highways: Survival of CTCs during cancer spread

### Circulation

After departing from the primary site, CTCs must adapt to the various forces of the metastatic cascade and changes in the TME through dynamic, non-hereditary modifications. It was found that fluid shear stress damage can stimulate mesotrypsin’s cleavage of protease-activated receptor 2’s N-terminal inhibitory domain, which in turn activates the Gαi protein and the Src-ERK/p38/JNK-FRA1/cJUN axis to turn on the expression of EMT markers and promote survival of CTCs instead of mediating apoptosis.^139^ Co-culture of CTCs with macrophages reveals that macrophages promote PCacell EMT plasticity. Mechanically fit CTCs guided by TAMs acquire an intermediate E/M state, characterized by flexibility and adhesiveness, resisting shear stress and enabling protective cell clustering.^140^ CTCs will be attacked by immunocytes, nevertheless, it is still largely unknown how successfully transferred CTCs escape immune surveillance. In human PDAC, through the immune checkpoint molecule pair HLA-E:CD94-NKG2A, CTCs keep interacting with NK cells to shield themselves from NK-mediated immune surveillance, according to cell-interaction studies.^141^ In PCa-related genes, significant silencing of gene clusters involved in CD1 genes, which participate in lipid antigen presentation to NKT cells, and interferon-induced genes associated with IFI16, involved in innate immunity, can be observed. This silencing favors the prevention of anti-tumor immune system activation, thereby protecting the survival of CTCs.^142^ CCL5 and CXCL5 mediate immune evasion by CTCs, enhancing their survival against immune surveillance. Upregulated expression of these chemokines by CTCs promotes recruitment of Tregs and neutrophils, dampening anti-tumor immune responses and facilitating CTC intravascular survival.^143^ Concurrently, immune cells located in secondary sites can interact with CTCs, facilitating the targeted transportation of CTCs that evade immune attacks to specific organs. For instance, evidence suggests that neutrophil extracellular traps (NETs) containing DNA, produced by neutrophils in the liver or lungs, act not only as traps for cancer cells but also as chemokines. These NETs bind to CCDC25 on the surface of CTCs, triggering the ILK-β-parvin-RAC1-CDC42 cascade, enhancing cell motility and promoting metastasis to the liver and lungs.^144^

Clusters of cells migrating collectively from primary tumors, comprising various cell types, appear significantly more effective than individual cancer cells at forming distant metastases.^145^ When TCs enter the bloodstream, it can induce invasive EMT and protect CTCs from shear-induced cell membrane damage and NK-induced cell death by releasing growth factors and small molecules to induce the formation of platelet-TC aggregates.^146^ Experimental evidence indicates that direct platelet adhesion induces upregulation of the inhibitory checkpoint CD155 in cancer cells via the FAK/JNK/c-Jun cascade, enabling evasion of NK cell cytotoxicity. This process is significantly associated with shortened progression-free survival (PFS) and overall survival (OS) in HCC patients.^147^ Moreover, platelets can efficiently transfer lipid, protein, and RNA structural components to TCs through mechanisms such as direct contact, internalization, or via EVs. This educational interaction educates TCs to acquire highly dynamic and invasive phenotypes.^148^ And intracellular bacteria carried by CTCs can also enhance the durability of CTCs against fluid shear by regulating host-cell actin network.^149^ Furthermore, in vivo research indicates that in heterotypic TC clusters, low-motile cancer cells may be transported by mesenchymal stromal cells or cancer-associated fibroblasts in a Rac-dependent manner, thereby accelerating the pace of metastatic dispersion.^145^

## Colonization frontiers: Settlement of metastatic tumor cells at the secondary sites

### Extravasation

#### Extravasation process of CTCs

Extravasation is a critical event involving the sequential process of cancer cell arrest on the endothelium, transendothelial migration, and subsequent invasion into the subendothelial ECM of distant tissues.^150^ Factors such as vascular endothelial contraction, injury, gap formation, and basal membrane expansion or damage significantly influence the extravasation process at secondary sites. Experimental observations have revealed the involvement of various immune cells in regulating vascular permeability. For instance, IL-22 derived from iNKT17 cells acts on endothelial cells (ECs) by inducing endothelial aminopeptidase N, promoting endothelial permeability and cancer cell migration.^151^ Matrix metalloproteinase 9 from monocytes within PMNs can also facilitate cancer cell extravasation by disrupting endothelial tight junctions.^152^ Furthermore, in obese mouse models, ROS produced by neutrophils have been found to increase the formation of NETs and weaken endothelial junctions. This impairment of neutrophil-dependent vascular integrity enhances the influx of TCs from the peripheral circulation, facilitating metastasis.^153^

During extravasation, there exists intricate crosstalk between TCs and other constituents such as ECs, leading to changes in functional behavior that promote extravasation. For instance, in spontaneous lung metastasis, the binding of amyloid precursor protein expressed by TCs to death receptor 6 triggers the necroptotic pathway, resulting in necroptosis of ECs and subsequently facilitating TC (TC) extravasation and metastasis.^154^ Membrane-bound metalloproteinase ADAM17 on ECs is also identified as a significant regulator of necroptosis, representing a potential target for anti-metastatic and late-stage cancer therapies.^155^ In CRC, enhanced adhesion between TCs and ECs is associated with upregulation of intercellular adhesion molecule 1 induced by nuclear Fusobacterium nucleatum.^156^ Additionally, secretion of C-C motif chemokine ligand 2 (CCL2) by astrocytes in the brain can act on type 2 C-C chemokine receptor (CCR2) on cancer cells, promoting their chemotactic and chemokinetic properties.^157^ In osteoblastic PCa cell lines, the circadian rhythm regulator, melatonin, inhibits FAK, c-Src, and NF-κB transcriptional activities via the melatonin MT1 receptor, effectively suppressing the expression of integrin α2β1, impacting the interaction between TCs and matrix components, and facilitating TC migration.^158^ Comparative proteomic studies have also revealed upregulation of CLIC1 expression in HCC, which recruits PIP5K to the plasma membrane leading to the generation of phosphatidylinositol 4,5-bisphosphate (PIP2)-rich microdomains, inducing integrin formation, participating in mediated cell-matrix adhesion, and cytoskeletal extension.^159^ Presently, most studies focus on the impact of single molecular targets on cancer cell extravasation function. However, the complex interplay involved in TC extravasation encompasses various cell types and signaling pathways, not entirely describable by a single target. For example, researchers using organotypic microfluidic models to study cancer cell and vascular interactions observed that the upregulation of multiple secretory factors and their combined effects impair vascular barrier function, influencing tumor extravasation behavior. Combined therapeutic inhibition of these factors may help slow the metastatic process.^160^ Moreover, the contractility of endothelial myosin and the mechanical properties of the subendothelial matrix also influence the extravasation capacity of TCs, as the protrusions rich in actin produced by cancer cells generate pushing and pulling forces that initiate and propel extravasation, with successful migration dependent on the force exerted by the endothelium.^161^ While endothelial-generated forces contribute to prolonged intercellular adhesion, excessive force can lead to adhesion detachment and rupture,^162^ highlighting the significance of endothelial subendothelial matrix mechanics and endothelial myosin contractility in influencing TC extravasation.

The cerebral vasculature, with its intricate structure, ensures adequate blood perfusion to meet the brain’s high energy demands.^163^ Circulating cancer cells must initially undergo permanent arrest within cerebral microvasculature to establish brain colonization, yet the key factors in this process remain unclear. Studies have found increased TC adhesion in larger vascular curvature regions, suggesting that prolonged tumor residence time under low velocity and wall shear stress accelerates the molecular features of metastatic potential.^164^ Furthermore, cancer cell-derived tissue factors can mediate thrombin-induced local activation of plasma clotting in the brain, leading to clot formation within cerebral microvessels, embedding numerous cancer cells within extensive clots, enabling prolonged stasis and enhancing the likelihood of successful extravasation.^165^ Thus, the synergistic interaction between platelets and the plasma coagulation system is crucial in promoting tumor dissemination. As such, anticoagulant therapy could become a significant candidate for future clinical trials aimed at preventing brain metastases.

#### Immunological viewpoints on characteristics of PMN associated with organ-specificity

The PMN is a specialized microenvironment prepared to host CTCs in specific organs. It consists of unique resident cell types, ECM components, and infiltrating cell populations. The variety of cell types and intricate interactions have conceptualized PMN.^9^ PMN manifests key attributes such as thrombosis, alterations in vascular permeability, ECM remodeling, and anomalous immunosuppressive inflammatory changes.^10^ The orchestration of organ-specific metastasis hinges on PMN formation, a process is usually guided by EVs, including microvesicles, exosomes, and large cancer vesicles released from malignant cells.^11^ Among these, exosomes from tumors circulate in the bloodstream, carrying inflammatory factors, PD-L1, and other compounds that can suppress the immune system, thus creating an immunosuppressive, inflammatory microenvironment favorable to the tumor within the PMN.^12^

Thrombus formation represents an early characteristic of PMN, fostering subsequent vascular dysfunction, ECM remodeling, inflammation, and immune suppression processes. Involvement in the early recruitment of PMN intratumoral macrophages and functional suppression of NK cells are critical for both PMN formation and CTC seeding.^166^ Furthermore, research reveals that low-density lipoprotein, closely associated with thrombus formation, exerts a pro-metastatic influence and associates with EVs derived from cancer cells.

Increased vascular permeability, a hallmark of PMN formation, correlates with heightened metastatic burden. Various immune cells participate in this process, promoting disruption of vascular integrity and hindering vascular normalization, leading to extensive immune cell and cancer cell extravasation into secondary tissues. For instance, NETs generated by neutrophils anchor onto vascular walls, releasing neutrophil elastase that disrupts vascular integrity through proteolytic cleavage, contributing to vascular instability and cancer-associated thrombosis.^167^ Research has also identified an independent pathway for vascular niche formation mediated by metastasis-associated macrophages. In this pathway, tenascin C derived from cancer-activated macrophages stimulates lung ECs via the secretion of NO and TNF, initiating the formation of a vascular niche.^168^ Pericytes, integral components of capillary walls, participate in fostering the aforementioned processes. Moreover, bidirectional crosstalk between pericytes and TAMs induces M2 phenotype macrophage infiltration and polarization, promoting tumor angiogenesis and facilitating the establishment of PMN conducive to hematogenous metastasis.^169^

ECM remodeling stands as one of the earliest, fundamental, and most significant precursors to metastasis in secondary organs, constituting a decisive feature in PMN development. Fibroblasts, resident cells, and immune cells participate in this remodeling process.^170^ For instance, in HCC lung metastasis, the recruitment of CD11b + /CD45+ bone marrow-derived cells (BMDCs) to lung tissue is driven by the release of Lysyl Oxidase-like 2 by HCC cells, alongside significant upregulation of MMP9 and fibronectin expression in lung fibroblasts. These factors collectively regulate ECM remodeling within the PMN.^171^ For instance, lipopolysaccharide binding protein derived from gastric cancer (GC) activates NF-κB in a TLR4-dependent manner, promoting the secretion of TGF-β1 by intrahepatic macrophages, subsequently activating HSCs, leading to increased ECM deposition in the liver and coordinating the formation of fibrotic PMN within the liver.^172^

Alongside ECM remodeling, the modulation of innate and adaptive immune cell function in secondary organs is crucial for the formation and evolution of PMNs, fostering a persistent inflammatory yet immunosuppressive microenvironment. Experimental findings indicate that tumor-derived extracellular vesicles (TEVs) are captured by host cells prior to the formation of PMNs. Differential gene upregulation observed in non-TEV-captured cells compared to TEV-captured cells suggests that TEV capture within PMNs can induce dynamic changes in inflammatory gene expression.^173^ Furthermore, TCs or stromal cells can release pro-inflammatory cytokines via autocrine and paracrine pathways, recruiting BMDCs and fostering the formation of an inflammatory environment, which gradually evolves into a pro-tumoral microenvironment as PMNs progress.^174^ Pharmacological or therapeutic interventions targeting the STING-TBK1-IFNβ axis can prevent the progression of inflammatory PMNs, thereby effectively inhibiting lung metastasis.^175^

#### Elucidating PMN formation in certain organs via an immunological insight

##### Neutrophils and NETs in breast cancer lung metastasis

Exosomal RNA influences metabolic reprogramming and cytokine secretion of target cells in lung tissue, facilitating neutrophil recruitment, immune-suppressive phenotype conversion, and NETs generation. For example, a recent study suggests that exosomal RNA from BC can activate TLR3 and its pivotal transcription factor IRF3 in alveolar epithelial cells. This activation boosts the promoter activity of HAO1, leading to its expression and causing an overproduction of oxalate. The accumulation of pulmonary oxalate subsequently triggers the formation of NETs by activating NADPH oxidase.^176^ In triple-negative breast tumors with elevated Lin28B expression, low levels of let-7 microRNAs in Lin28B-positive exosomes modulate IL-6 and IL-10 production in lung fibroblasts. This modulation facilitates neutrophil recruitment and polarization towards an N2 phenotype. These N2 polarized neutrophils then alter the balance between immunostimulatory IL-12a and immunosuppressive IL-6 and IL-10, impeding the differentiation of CD4+ T cells into T helper cells (Th) 1. This results in insufficient activation of CTLs, an increased Th2 ratio, and overall immune suppression.^177^ Concurrently, Th2 cytokines such as IL-4/IL-13 were noted for activating local mesenchymal stromal cells in lung PMN to upregulate C3 expression, further promoting neutrophil recruitment and NET formation via the upregulation of the STAT6 signaling pathway, facilitating lung PMN establishment.^178^

Epidemiological and clinical research indicates that environmental pollutants adversely impact the body’s innate and adaptive immune system. Prolonged exposure accelerates lung function decline and correlates with a notable rise in lung cancer incidence and mortality. Despite this, limited experimental studies have explored the mechanisms underlying pollutant exposure in promoting lung metastasis progression. According to research, nicotine, the primary addictive component in smoke, can triggers STAT3-dependent N2-polarization of neutrophils. These N2-type neutrophils exhibit selective colonization in the lungs of tumor-free mice and release lipocalin 2 in a paracrine manner, contributing to the establishment of a conducive microenvironment for subsequent TC implantation.^179^ Similarly, particulate matter, a main component of air pollutants, induces TRAF6 accumulation via ROS-triggered, autophagy-dependent degradation of TRIM37 in alveolar epithelial cells, leading to increased NFκB pathway activation and enhanced chemokine production. This process will also foster the formation of a lung PMN through neutrophil recruitment.^180^ Moreover, prior research has identified the TLR3 pathway as prominently modified in premetastatic alveolar epithelial cells, with elevated expression strongly linked to chronic inflammation induced by smog.^176^

**Fig. 6:**
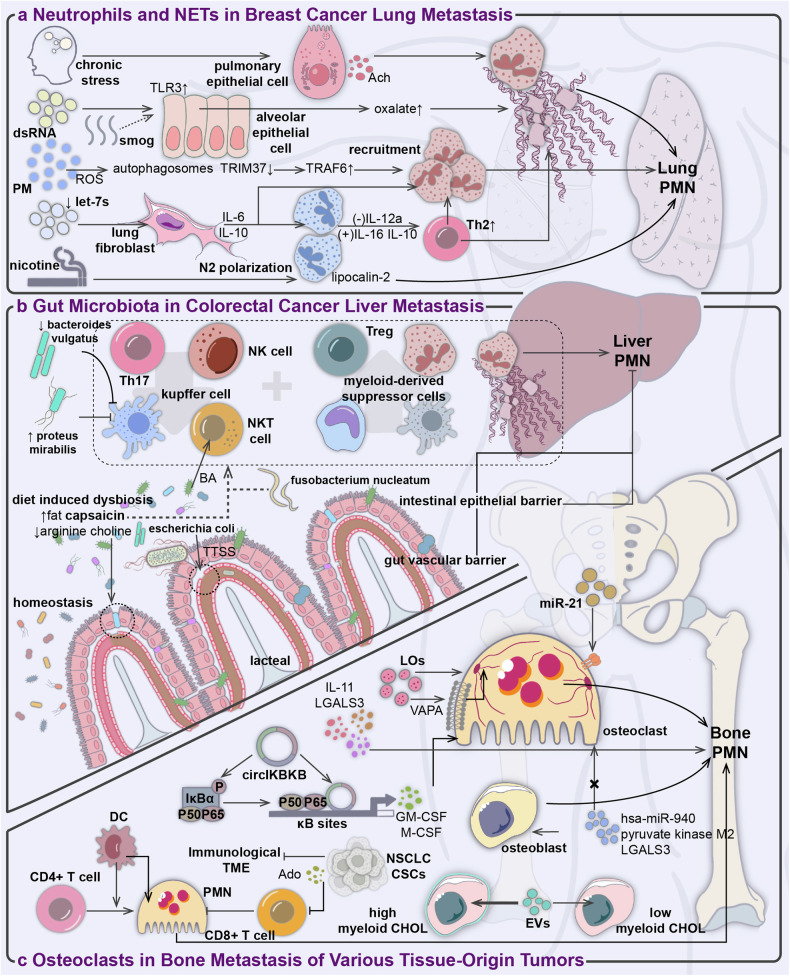
Perspectives from immunology on the organ-specificity of PMN formation. **a** Chronic stress activates pulmonary epithelial cells to secrete ACh, promoting lung NETs production; extracellular vesicle RNA of BC can upregulate TLR3 expression in alveolar epithelial cells, thereby triggering NETs formation. Elevated TLR3 pathway expression is also associated with smoke-induced chronic inflammation. Air pollutants induce autophagy-dependent TRIM37 degradation in alveolar epithelial cells, promoting neutrophil recruitment. Reduced let-7s in Lin28B EVs also participate in regulating neutrophil recruitment. Furthermore, low let-7s in EVs and nicotine promote lung PMN formation by fostering neutrophil N2 polarization. **b** The gut-liver axis consists of the intestinal epithelial barrier and the GVB, which collectively protect the liver from invasion by commensal or pathogenic microbes from the intestine. Alterations in the gut microbiota induce immune responses in the liver, mediated by the recruitment of MDSCs and Tregs, and reduction in Th17, NK cells, KCs, and NKT cell infiltration. This process is influenced by dysbiosis induced by various pathogenic bacteria and dietary factors. **c** Tumor-secreted factors directly or indirectly influence OC activation, leading to the formation of bone PMN. In BC bone metastasis, tumor-derived EVs containing miR-21 regulate the expression of programmed cell death 4, impacting PMN formation. Upregulation of circIKBKB significantly enhances IκBα phosphorylation, inducing the expression of GM-CSF and M-CSF, effectively promoting osteoclastogenesis. In HCC bone metastasis, large oncosomes (LOs) facilitate cellular cytoskeletal rearrangement and OC formation, while cytokines from HCC cells also contribute to bone metastasis. Additionally, EVs secreted by PCa induce premetastatic osteoblastic lesions, and cholesterol homeostasis in BM stromal cells plays a gatekeeping role in regulating PCa-promoting EV signal transduction. The immune TME plays a crucial role in bone PMN formation before bone colonization and is sensitive to various extracellular substances

Research indicates that sympathectomy in mice using 6-hydroxydopamine reduces MDSC recruitment and pulmonary metastasis. Additionally, in a tumor-bearing mouse metastasis model, there are observable neuro-immune cell interactions, collectively suggesting a potential role of the sympathetic nervous system in priming PMNs in the lungs.^181^ However, prior studies have mainly concentrated on the acute stress-induced activation of the systemic sympathetic nervous system and the release of stress-induced hormones. Within BC mice, chronic stress promotes neutrophil infiltration into the lungs via the CXCL2-CXCR2 axis. It also activates the acetylcholine pathway in lung epithelial cells with neuroendocrine functions, boosting lung NETs production through acetylcholine secretion and facilitating NETotic neutrophils in capturing cancer cells.^182^ Several studies mentioned above demonstrate that various tissue-specific cells are involved in the formation of lung PMNs, reshaping the local microenvironment and promoting distant metastasis. (Fig. 6a).

##### Gut microbiota in colorectal cancer liver metastasis

The venous blood from the gastrointestinal tract directly reaches the liver through the portal vein, exposing the liver to metabolites and components from the intestines through the portal circulation. Consequently, the metastasis of CRC TCs to the liver via venous blood appears to be the most likely hematogenous route.^183^ Recent research highlights a potential link between the pre-metastatic liver microenvironment and the transit of intestinal microbiota and their metabolic byproducts through the gut-liver axis. (Fig. 6b) The gut-liver axis, consisting of the intestinal epithelial barrier and the gut vascular barrier (GVB), collaboratively safeguards the liver from invasion by commensal or pathogenic microorganisms in the intestinal tract. Notably, compromised epithelial integrity is a common observation in both CRC patients and animal models.^184^

Escherichia coli C17 has been demonstrated to breach the GVB directly through a TTSS virulence factor-dependent mechanism, relocating to the liver and facilitating PMN maturation.^185^ GVB damage is also observed in specific dietary conditions, such as HFDs or those deficient in arginine and choline, with alterations dependent on diet-induced modifications of the microbial community.^186^ For example, capsaicin enhances intestinal barrier permeability by modulating the composition and abundance of gut microbiota. Extended exposure to capsaicin in a murine CRC model leads to an elevation in hepatic neutrophils, macrophages, and monocytes, alongside a marked decrease in natural killer T (NKT) cell populations.^187^ Neutrophils utilize mechanisms like phagocytosis, oxidative bursts, antimicrobial mediator release, and the formation of NETs to eliminate intestinal toxins. However, excessive NET accumulation in the liver may induce damage and suppress immune cell activity, promoting tumor metastasis.^188,189^ In vivo studies indicate that NKT cells selectively suppress tumors in the liver. NKT cell accumulation is regulated by gut microbiota-mediated conversion of primary bile acids to secondary bile acids. These findings collectively suggest that prolonged intestinal dysfunction leads to shifts in microbial composition and subsequent alterations in the immune microenvironment of the liver.^190^ Studies on Fusobacterium nucleatum-treated mice demonstrated a notable elevation in plasma levels of pro-inflammatory cytokines, including IL6, IL12, CXCL1, MCP-1, TNF-α, and IFN-γ. Furthermore, the liver’s immune response was modulated through the recruitment of MDSCs and Tregs and reduced infiltration of NK cells and Th17 cells.^191^ Another investigation highlighted the crucial role of Proteus mirabilis abundance and reduced Bacteroides vulgatus quantity in CRC liver metastasis, linked to diminished kupffer cells (KCs), the predominant macrophage population in liver sinusoids.^192^ The gene encoding EV angiogenesis-like protein 1 was identified in reprogramming KCs, hindering vascular leakage in liver PMN, and attenuating CRC liver metastasis.^193^

##### Osteoclasts in bone metastasis of various tissue-origin tumors

The stromal components in both the primary TME and distant organs dictate tumor metastatic tropism. Bone, a dynamic tissue and a frequent site for cancer metastasis, undergoes regulation through osteoclast (OC)-mediated bone resorption and osteoblast-mediated bone formation, harmonizing remodeling equilibrium.^194,195^ Tumor-secreted factors influence OC activation, either directly or indirectly, resulting in bone matrix resorption and the establishment of a PMN. Bone matrix degradation releases growth factors, facilitating colonization and expansion of bone metastatic TCs.^196^ (Fig. 6c) In BC tissues with bone metastasis, the upregulation of circ IKBKB significantly promotes IKKβ-mediated phosphorylation of IκBα, suppressing the IκBα negative feedback loop. This promotes NF-κB activation towards various bone remodeling factors’ promoters, inducing the expression of M-CSF and granulocyte-macrophage colony-stimulating factor. This reinforces the NF-κB activation feedback loop, effectively promoting osteoclastogenesis and BC bone metastasis.^197^

Tumor-derived exosome and EVs serve as vital messengers in regulating OC activity and substantially contribute to PMN formation. For example, BC cell-derived exosome containing miR-21 modulate Programmed Cell Death 4 expression, influencing PMN formation.^198^ In a hepatic cancer bone metastasis model, large oncosomes, atypical EVs, play a crucial role in promoting osteoclastogenesis by delivering VAMP-associated protein A to OCs’ plasma membrane, facilitating cytoskeletal reorganization and OC formation.^199^ Factors derived from HCC cells, such as IL-11, lectin galactoside-binding soluble 3 (LGALS3), and long non-coding RNA H19, also contribute to HCC bone metastasis and associated skeletal complications.^200^ Notably, PCa-secreted EVs containing hsa-miR940, pyruvate kinase M2, and LGALS3 influence osteoblast differentiation, inducing premetastatic osteoblastic lesions without inhibiting OC differentiation. Thus, TC factors and EVs from diverse origins may modulate the bone microenvironment through distinct mechanisms, showcasing varied biological activities.^201,202^ Despite significant progress in understanding pro-metastatic EVs in PMN formation, further exploration is required for intrinsic factors in recipient cells that can modify the reception and transduction of pro-metastatic EV signals. For example, cholesterol levels are significantly elevated in PCa bone metastatic lesions compared to healthy bone, indicating that cholesterol homeostasis in BM stromal cells may serve as a critical regulator of pro-metastatic EV signal transduction and influence distant bone metastasis.^203^

Furthermore, prior to bone colonization, the immunological TME plays a crucial role in shaping the PMN in secondary organ site and in the metastasis development itself.^204^ Research indicates that RANKL + CD4+ T cells specific to the 4T1 BC cell line reach the BM and establish PMN before metastatic cell arrival. Additionally, CD8+ T cells from 67NR tumor origins contribute to bone homeostasis, while dendritic cells create a positive feedback loop inducing osteolytic changes to maintain a phenotype promoting osteoclastogenesis in T cells. OCs can also initiate T-cell responses, forming a feedback loop that collectively regulates bone tissue PMN formation.^205^ The immune TME involved in PMN formation is susceptible to various extracellular substances. In NSCLC, a subset of CD133/CXCR4+ CSCs initiating bone metastasis expresses enzymes CD38, PC-1, and CD73 along non-classical adenosine pathways, producing high levels of Ado. This process downregulates the inhibitory receptors A1R and A3R while upregulating A2AR and A2BR, directly impairing anti-tumor immune responses and contributing to T-cell suppression.^206^

### Colonization

#### Dormant stage of DTCs

Typically, these disseminated tumor cells (DTCs) will restore their epithelial phenotype via Mesenchymal-epithelial transition (MET) upon reaching a conducive PMN, facilitating colonization and proliferation into a new tumor. However, some may transiently retain their mesenchymal phenotype, rendering them dormant TCs.^207^ During the dormant cancer cell life cycle, cancer cells initially occupy ecological niches in the secondary sites, undergo G0-G1 cell cycle arrest and cell reprogramming after binding to receptors in the niches, and then activate immune evasion mechanisms to adapt to the niches and enable long-term dormancy.^31^

In BC bone metastasis, utilizing a robust 3D indirect coculture model of BC cells with BM niche cells, researchers identified that the BM niche induces dormancy in DTCs through cell-cell and cell-ECM interactions, with a key role played by autophagy in survival.^208^ This finding underscores the dual regulation of cancer cell dormancy, involving both intrinsic cellular mechanisms and extrinsic control by the niche. The interaction among the immune system, cancer cells, and tissue-specific stromal cells is a critical factor in this process.^151^ Nevertheless, there are still few precise insights into the cells and molecules that mediate this communication. It was found that transcription factor ZFP281, absent in advanced primary tumors and dominant metastasis, act as an inducer of mesenchymal- and primed pluripotency-like programs and locks early DTCs in a long-term dormant state rather than outgrowth by preventing the acquisition of epithelioid proliferative programs.^209^ And in a model of BC lung metastasis, a rich population of immune cells exhibiting both pro-inflammatory and anti-tumor phenotypes can be observed in the dormant lungs of mice. Dormant DTCs actively recruit N1 neutrophils with potent local anti-tumor immune capabilities, thereby suppressing metastatic extrinsic growth.^210^ Furthermore, research has revealed the involvement of the primary TME in the induction of dormancy phenotypes in DTCs. For instance, TCs acquire the dissemination and dormancy program through interactions with macrophages near TME of metastasis portals within the primary tumor. This programming imparts effective extravasation capabilities to TCs, along with the ability to resist proliferative chemotherapy through dormancy, leading to the formation of metastatic foci.^211^

#### Initiation of apparent macrometastases by MICs

Tumor metastasis, characterized by a series of sequential steps, exhibits notable inefficiency. Only cancer cells capable of successfully completing all essential steps can lead to detectable metastases.^150^ Subsequently, dormant cancer cells re-exhibit a proliferative phenotype in response to ecological niche alterations, including sufficient nutrient accumulation, the establishment of immune escape microenvironments, or stimulatory signals from neovascular growth. Successful metastasis-initiating cells (MICs) necessitate intricate interactions within their TME for the development of clinically evident macrometastases.^31,57^

The metastatic cascade involves a sequential activation of EMT molecular programs, where hybrid E/M cancer cells traditionally shift towards a predominantly epithelial state to establish metastases. However, studies in triple-negative BC genetically engineered mouse models and patients revealed that metastases express a diverse range of epithelial, hybrid E/M, and mesenchymal markers. This indicates that MET may not be universally required for MIC metastasis, highlighting heterogeneity both among and within metastases in the same individual. Thus, multiple metastatic molecular programs may operate concurrently.^212^ Furthermore, the cellular components in microenvironment of secondary sites, modulated by factors like patients’ condition and TCs, facilitate the establishment of a conducive ecological niche for the robust proliferation of MICs. Notably, preliminary findings indicate that WNT5A acts as a latent activator in melanoma lung metastasis. Age-induced programming changes in lung fibroblasts result in heightened secretion of its antagonist sFRP1 and other age-related soluble factors, fostering efficient metastatic growth.^213^ Beyond secondary site stromal cells, the crucial involvement of immune cells in this process cannot be overlooked. For example, tissue-resident and recruited macrophages crucially regulate metastatic growth, with mechanisms varying across secondary organs and cancer types. In bone metastasis, TC-induced OCs secrete IL-19, a ligand of IL-20RB, initiating downstream JAK1/STAT3 signaling in TCs, thereby enhancing TC growth in bone.^214^ Concurrently, macrophages derived from Ly6C + CCR2+ inflammatory monocytes, characterized by elevated CD204 and IL4R expression, play a crucial role in the IL4R-dependent amplification of BC bone metastases.^215^ In a similar manner, the tumor-secreted protease cathepsin C regulates neutrophil recruitment and NETs formation, facilitating the degradation of microenvironmental heterogeneous signals, including platelet-reactive protein-1. This process contributes to the promotion of BC lung metastasis.^216^

However, the treatment of metastatic tumors remains challenging, with patient prognosis largely dependent on early diagnosis of the primary tumor. Tumor biomarkers, substances produced by tumors or the body’s response to tumors during their occurrence or progression, have demonstrated critical and promising value in screening, early diagnosis, and prognosis.^217^ The use of clinically established screening biomarkers, such as alpha-fetoprotein (AFP) and prostate-specific antigen (PSA), along with circulating nucleic acid biomarkers including cell-free DNA (e.g., circulating tumor DNA (ctDNA), cell-free mitochondrial DNA, and cell-free viral DNA) and cell-free RNA, is crucial for the early diagnosis of tumor metastasis.^218^ Furthermore, in response to the increasing demand for early and precise cancer detection, the development of highly sensitive and specific biosensors and their integration with traditional detection methods is crucial.^219^ For instance, ultrasound combined with AFP is recommended for monitoring HCC, with PIVKA-II considered valuable for detecting HCC in AFP-negative patients and potentially predicting microvascular invasion, aiding in liver transplant selection.^220^ Additionally, the combined biomarker panel of DCP (des-γ-carboxy prothrombin) and AFP-L3 strongly predicts HCC recurrence post-liver transplantation.^221^ Moreover, studies have found that genetic determinants of constitutive, non-cancer-related PSA variants may enhance screening efficacy, reduce false negative prostate biopsies, and better predict aggressive prostate cancer.^222^ Currently, there is growing emphasis on the prognostic value of tumor biomarkers for predicting individual patient outcomes, treatment responses, and monitoring therapeutic efficacy. For example, observations indicate that HR + /Human Epidermal Growth Factor Receptor 2- (HER2-) metastatic BC patients with PIK3CA mutations have poorer prognosis and resistance to chemotherapy, whereas TNBC patients with PIK3CA mutations have better OS, thus emphasizing the significant impact of hormone receptor and HER2 expression status and PIK3CA mutations on the prognosis of metastatic BC patients.^223,224^ Additionally, soluble MUC1 levels serve as prognostic predictors for early and advanced BC, where pre-chemotherapy MUC1 has no prognostic value for lymph node-negative patients but correlates significantly for lymph node-positive patients, potentially serving as a suitable tool for identifying adverse prognosis in lymph node-positive groups.^225^ In the era of targeted therapy based on genomic alterations, determining tumor genomic status before initiating systemic treatment is recommended. For instance, in metastatic CRC, detecting KRAS and NRAS mutations, BRAF^V600E^ mutations, NTRK fusions, HER2, ERBB2, microsatellite instability, and/or mismatch repair status facilitates selecting appropriate first-line treatment modalities.^226,227^ In BRAF^V600E^ metastatic colon cancer, whole exome sequencing reveals that loss-of-function mutations in WNT negative regulator RNF43 can predict improved response rates to BRAF/EGFR therapy and survival outcomes in microsatellite stable tumor patients, demonstrating novel predictive biomarkers that facilitate optimizing patient clinical management.^228^

## Principles for managing multi-phase tumor progression of the post-metastasis landscape

### Micrometastasis

DTCs preferentially lodge around organ-specific vasculature, with the majority cleared by the host immune system, while a minority of MICs survive, entering an immune-evasive dormant state, gradually acquiring organ-specific growth adaptation. This metastatic phase is often undetectable by conventional imaging or routine examinations but can be identified through histological or molecular biology methods, referred to as micrometastases. Targeting the eradication of micrometastases and reversing disease progression may significantly enhance patient prognosis.^229^ Surgery and chemotherapy are commonly employed effective treatment modalities for metastatic cancer patients, typically effective in treatment, yet paradoxically, tumor-killing mediates acute inflammatory cytokine and EV release, favoring the establishment of pre-metastatic niches, thus facilitating tumor dissemination and micrometastasis occurrence.^230^ Studies indicate that preoperative administration of nonsteroidal anti-inflammatory drugs like ketorolac and/or dexamethasone can block pro-inflammatory responses, activate endogenous resolution pathways, and inhibit immune checkpoints to enhance T-cell responses. This approach may assist in clearing micrometastases, reducing tumor recurrence, and achieving long-term patient survival.^231^ Additionally, pretreatment with the anti-PD-1 monoclonal antibody nivolumab has been demonstrated to significantly benefit the treatment of residual micrometastatic disease, even aiding in organ preservation, in high-risk bladder, esophageal, or GCs.^232^

In recent years, with advancements in molecular imaging technologies and a deeper understanding of CTC research, the prospects of identifying and tracking micrometastatic diseases have become increasingly crucial. For instance, Raman imaging has emerged as a powerful tool for cancer diagnosis and visualization of various biological processes. Researchers have discovered an IDT-BT polymer Raman probe suitable for in vivo imaging, successfully achieving intraoperative Raman imaging of micrometastases as small as 0.3 mm × 0.3 mm and enabling non-invasive microvascular imaging, assisting physicians in earlier detection of micrometastatic presence and subsequent interventions by observing microvascular structure and function.^233^ Moreover, through molecular flattening strategies, researchers have developed tunable thiophene polymer probes, aiding detectors to successfully bypass lipid signals around tissues, further advancing the development of high-precision intraoperative Raman imaging.^234^ Additionally, near-infrared imaging is regarded as a promising method for biological imaging. Several near-infrared fluorescent probes have gained clinical approval. Studies have found that albumin, one of the most abundant proteins in plasma, has high binding affinity with various highly expressed albumin receptors. Based on this discovery, researchers have developed a fluorescence probe named IR1080 with enhanced micrometastasis tracking and anchoring capabilities, achieving high detection rates and delineation capabilities, serving as another efficient strategy for micrometastasis diagnosis.^235^ In the fields of magnetic resonance imaging (MRI) and positron emission tomography (PET)against BC-related micrometastases imaging, a growing number of efficient imaging agents are under development, exhibiting higher diagnostic accuracy and assisting in accurately identifying micrometastatic patients. For example, in hepatic metastasis from uveal melanoma, a collagen-targeted protein contrast agent (ProCA32.collagen1) has demonstrated early sensitivity to hepatic micrometastases and two distinct tumor growth patterns, capable of detecting lesions as small as 0.144 mm².^236^ In a model of lung metastasis from osteosarcoma, tumor-derived exosomes as targeted imaging agents can be detected non-invasively via PET for micrometastatic lesions, with a sensitivity of up to 2-3 millimeters.^237^ Therefore, further research and development are expected to greatly promote the early non-invasive detection of micrometastatic foci in secondary sites.

Multiple large-scale clinical studies have confirmed the prognostic value of detecting and characterizing CTCs in various types of cancer. To gain a deeper understanding of the potential mechanisms underlying metastatic cascades, it is crucial to study CTCs that play important roles in the successful establishment of distant metastases.^238^

These cells can serve as monitoring biomarkers or key elements in blood-based non-invasive liquid biopsies, providing crucial diagnostic and treatment-related information to guide personalized treatment decisions.^239^ Research indicates that CTCs in multicellular clusters, which exhibit stem cell properties, have a 20-100-fold higher metastatic potential compared to individual cells.^240^ In BC patients, dynamic alterations in CTCs involving low sialylation or ST6GAL1 defects promote the seeding of CTCs in cluster form and enable evasion of paclitaxel therapy. Hence, dynamic changes in both individual and clustered CTCs impact overall tumor survival rates, and the ST6GAL1 glycoprotein substrate PODXL may be a favorable candidate target against BC-related micrometastases.^241^ The presence of ctDNA also holds significant prognostic value in distant metastatic recurrence of various tumors. For instance, after bladder resection, ctDNA analysis correctly identified all patients with recurrent metastases during disease monitoring.^242^ In HR-positive/HER2-negative BC patients, ctDNA positivity is associated with a reduced rate of distant recurrence-free survival, whereas ctDNA negativity is associated with improved prognosis.^243^ Therefore, monitoring ctDNA may potentially replace standard care tissue biopsies based on DNA biomarker assessment, potentially improving clinical management in predicting the future recurrence of many cancers.^244^

The extravasation process of DTCs upon reaching secondary sites depends on effective angiogenesis, with the angiopoietin/Tie pathway being a critical regulator of angiogenesis.^245^ Recently, human antibodies targeting the Tie1 receptor have demonstrated clinical potential in inhibiting the extravasation of TCs to organs like the lungs, without negatively impacting immune cell infiltration. This approach may serve as a selective antagonist therapy for micrometastases.^246^ In the brain, abnormal angiogenesis is associated with abnormal changes in the blood-brain barrier, facilitating the settlement and spread of TCs in the brain. However, the blood-brain barrier permeability at brain micrometastatic sites poses a significant barrier to effective treatment. Newly developed TNFR1 selective agonist variants selectively enhance blood-brain barrier permeability at micrometastatic sites, while preserving the integrity of the rest of the brain vasculature. This strategy opens up a new therapeutic avenue for poorly prognosed cases of brain metastases, providing previously non-existent treatment possibilities.^247^ Additionally, DTCs successfully extravasating to secondary sites may undergo one of four fates: death, cell dormancy, dormant micrometastasis, or invasive growth mediated by ECM characteristics.^248,249^ Therefore, gaining a deeper understanding of how specific ECM characteristics induce and regulate the fate of DTCs may aid in the development of new therapeutic strategies targeting the ECM, thus advancing the development of future therapeutic approaches to delay or prevent micrometastases.^250^

### Oligometastasis

In the early stages, most patients with solid tumor metastases are considered incurable. However, some surgical reports indicate that a minority of patients experience long-term disease-free survival after undergoing resection of the primary tumor metastatic foci.^251^ Furthermore, well-standardized systemic treatments have demonstrated increased efficacy in certain patients with metastatic or recurrent tumors.^252,253^ Since the concept of oligometastasis was introduced in 1995, this state is typically described as a dynamic clinical condition with limited metastatic spread. Patients demonstrate favorable survival outcomes through receiving local treatment.^254,255^ Oligometastasis is generally defined by the number of metastatic foci; for example, some current clinical trials set the limit at ≤3 or ≤5 metastatic lesions.^256^

However, studies have found that patients’ clinical risk stratification and tumor and host biological characteristics also influence the prediction of metastatic phenotypes to varying degrees, thus leading to the ongoing refinement of the definition of oligometastasis.^257–260^ Research findings indicate that factors such as age, Child-Pugh classification, and AFP levels in controlled primary HCC patients have statistical significance levels of 0.002, 0.004, and 0.019, respectively, considered important factors in measuring patient metastatic characteristics.^261^ Moreover, the histological subtype of the primary tumor, postoperative margin nature, lymph node involvement, patient’s overall condition, and disease-free interval also play significant roles in prognosis assessment.^262^ In some cases, tumor molecular features, particularly the molecular classification of metastatic tumors, are crucial for predicting whether patients will develop an oligometastatic phenotype. Some experimental observations suggest that the shorter the time from the primary tumor to the appearance of oligometastasis, the shorter the PFS of patients. It has also been demonstrated that due to differences in tumor activity of the primary lesion, metachronous oligometastasis has a better prognosis than synchronous oligometastasis.^255,261^ Furthermore, in a retrospective study of men with metastatic castration-sensitive prostate cancer (CSPC), TP53 mutations were found to be associated with the oligometastatic status of patients and an increase in the number of metastatic foci, while mutations in DNA double-strand break repair genes were associated with more metastatic foci.^263^ In CRC liver metastasis, amplification of the VEGFA gene accompanied by markers of stroma, stromal cells, and angiogenesis, or exclusive NOTCH1 and PIK3C2B mutations associated with E2F/MYC activation, can be observed in adverse prognosis subtypes.^264^ Additionally, some microRNAs, such as miR-200c, miR-487a-5p, and 14q32-encoded miRNAs, have been shown to potentially aid in distinguishing patients who benefit from metastasis-directed therapy (MDT).^265–267^ These findings suggest that tumor molecular subtypes serve as a supplement to clinical risk stratification, differentiating patient risk. Therefore, integrating tumor genetics may further refine the current definition of oligometastasis. Besides tumor intrinsic biological features, factors such as interferon, CD8+ T cells, and metabolites from intestinal microbiota are associated with the tolerance response to local radiotherapy,^259,268^ underscoring the importance of systemic macroenvironment in risk stratification for the oligometastatic phenotype.

In recent years, significant advancements in systemic therapy have markedly improved the prognosis of metastatic cancer patients,^269^ yet disease progression often occurs following initial response. Technological progress has expanded the scope of local ablative therapies, thus combining local treatment with systemic therapy may offer long-term survival for metastatic cancer patients.^270^ Randomized trials in oligometastatic PCa patients have demonstrated that MDT +/− systemic drug therapy can enhance treatment efficacy, providing novel therapeutic modalities.^271^ In patients with oligometastatic NSCLC, the addition of stereotactic body radiotherapy to standard systemic therapy resulted in significantly improved treatment efficacy, with a more than fourfold increase in PFS compared to the group receiving standard systemic therapy alone.^272^ However, as tumor burden increases, the efficiency of MDT and systemic therapy is compromised, highlighting the importance of timely tumor debulking surgery as a crucial component of oligometastatic combined therapy.^273,274^ While local therapy benefits some metastatic tumor patients, it remains challenging, particularly in identifying patients most likely to manifest oligometastatic phenotypes. For instance, in PCa patients with elevated PSA post-maximal local therapy but negative clinical staging, PSMA-targeted PET-MR/CT detected cancer recurrence in 3/4 patients, with significant PSA reduction observed in half of the patients following regional targeted therapy, emphasizing the importance of enhancing imaging techniques for accurate diagnosis of oligometastatic disease.^271^ In summary, oligometastasis represents a transient state in the natural course of tumor progression, and early confirmation of oligometastatic disease diagnosis contributes to overall prognosis improvement for patients. Therefore, refining the definition of oligometastasis through integrating molecular mechanisms of tumor intrinsic metastatic potential, host systemic macroenvironmental alterations, and novel diagnostic technologies is advantageous for establishing a more effective diagnostic framework for managing metastatic cancer patients, thereby improving survival length and quality of life for many advanced cancer patients.

### Macrometastasis

As a consequence of certain local or systemic events, DTCs or micrometastatic deposits exit dormancy and initiate proliferation, leading to the development of actively growing and ultimately fatal large metastases.^275^ Early-stage metastatic tumors are typically managed with multimodal therapies, including chemotherapy and targeted therapies, depending on individual circumstances.^276^ However, as the disease progresses to an advanced stage with increasing numbers and widespread distribution of metastatic lesions, accompanied by locally uncontrollable manifestations, chemoresistance, and poor prognosis, treatment options become severely limited. Consequently, palliative care integrated with oncological treatment often becomes the standard approach for managing late-stage metastatic patients in clinical practice.^277^ In advanced CRC patients lacking effective systemic treatment options, therapies such as treatment with Futibatinib or combination immunotherapy with Durvalumab and Tremelimumab have shown significant associations with prolonged OS and PFS compared to placebo. Clinical trial results support Futibatinib as a novel oral treatment option globally.^278,279^ Additionally, to address unresectable diffuse peritoneal metastases and ascites formation in advanced CRC patients, the OxP/R848@PLEL hydrogel delivery system has been developed to effectively eradicate metastases and ascites, thereby prolonging patient survival.^280^ Furthermore, in cancer patients with musculoskeletal involvement, minimally invasive musculoskeletal interventions such as thermal ablation, neurolysis, and palliative injections, as local treatment options, have been shown to facilitate durable, timely, safe, and effective palliative care, gradually integrating into the clinical management of patients.^281^ Moreover, clinical trials have confirmed the effectiveness of stereotactic body radiation therapy in controlling symptoms in patients with painful spinal metastases.^282^ All of these underscore the importance of specialized and multidisciplinary involvement in palliative care for advanced cancer patients. In recent years, palliative care has developed into a multidisciplinary specialty, requiring further research to establish standards for interventions, refine care models, explore integrating primary palliative care with specialized oncology treatments, and tailor personalized care plans based on individual patient needs and settings, thus improving access to high-quality palliative care.^283,284^

## Exploring potential therapeutic strategies against tumor metastasis

### Surgical and interventional therapies

Tumor resection, utilizing surgical procedures to achieve tumor reduction, is often plagued by high rates of recurrence worldwide. Post-metastasis recurrence and dissemination are primarily attributed to tumor micro-metastases, changes in the immune microenvironment, and CTCs in the bloodstream.^285^ Besides intraoperative cancer cell dissemination, researchers have identified metastatic cancer cell invasion into adjacent brain tissue as associated with local recurrence and shortened OS in brain metastases, suggesting peritumoral invasion may be a key driving factor for postoperative tumor recurrence and metastasis.^286^ However, the role of secondary craniotomy in patients with locally recurrent brain metastases remains controversial. Comparisons with matched patients undergoing craniotomy for newly diagnosed brain metastases revealed that recurrence predicts shortened OS, but intracranial control remains unaffected, thus secondary craniotomy may be considered for patients with relatively favorable clinical features and good survival outcomes.^287^ In single-cell transcriptomic data of CRC liver metastasis patients, a unique population of TCs expressing the most adverse prognostic genes was identified. Observations in murine models showed that these residual cells after surgery lurk in the liver, giving rise to various cell types over time, ultimately leading to metastatic disease. Targeting and selectively eliminating this cell population may prevent metastatic recurrence, suggesting in-depth study of residual lesion cell dynamics and prediction and targeting of such cells may help avoid metastatic recurrence.^288^ For residual tumor tissue after surgery for metastatic tumors, combined therapy involving tumor excision and postoperative in-situ implantation presents a feasible strategy with significant clinical translational prospects. For instance, researchers designed an implantable sandwich-structured dual-drug reservoir, initially releasing combretastatin A4 phosphate to disrupt existing vasculature and inhibit neoangiogenesis, thereby cutting off the external energy supply to cancer cells, followed by the release of tigecycline to induce apoptosis under hypoxic conditions.^289^ Additionally, postoperative in-situ implantation of peroxide copper nanoparticles-loaded hydrogel systems has been shown to eliminate residual lesions through induced DNA damage, immunogenic TC death, and copper-induced death.^290^ Similarly, a novel photothermal fiber chitosan/polydopamine sponge also shows the potential to improve the immune microenvironment, while having the capability to ablate microlesions and enhance hemostasis, thereby reducing the likelihood of residual TC extravasation from multiple dimensions.^285^

In addition to distant metastasis, invasion of tumor tissue into surrounding lymph nodes is also associated with poor prognosis. Traditionally, lymphadenectomy during surgery is required to identify lymph node metastasis. However, randomized clinical trials related to endometrial cancer have shown that lymphadenectomy, independent of adjuvant treatment effects, does not improve patient survival rates. Therefore, sentinel lymph node biopsy may serve as a less invasive lymph node assessment strategy and may replace intraoperative lymphadenectomy in certain cases.^291^ The development of novel DROP-IN gamma probes has been found to effectively identify sentinel lymph nodes intraoperatively, with complementary optical confirmation of nodal localization through fluorescence imaging, greatly enhancing intraoperative detection accuracy.^292^ However, lymph node dissection remains the gold standard for lymph node staging, though there is ongoing debate regarding the optimal extent of dissection. A randomized controlled trial showed that expanding lymph node dissection did not reduce the biochemical recurrence of PCa to the expected level, providing more accurate pathological staging only in intermediate- and high-risk patients. Consequently, indiscriminate expansion of dissection scope is not recommended for precise lymph node staging; instead, strict adherence to extended templates should be maintained for intermediate- and high-risk patients.^293^ Additionally, for patients with lymph node metastasis, the anatomical pattern of lymph node involvement should be fully considered to select the appropriate salvage treatment (surgery or radiotherapy) to maximize the therapeutic effect.^294^

Transarterial chemoembolization (TACE) is a treatment method for blocking tumor blood supply by injecting embolic agents combined with chemotherapy drugs, recognized as a standard procedure for liver tumor therapy.^295^ However, TACE treatment still faces challenges. Firstly, successful TACE treatment urgently requires an embolic agent with therapeutic efficiency matching expectations. Inspired by the structural support characteristics of cell walls and defense mechanisms, researchers have developed a lignin-based embolic nanogel, exhibiting ideal mechanical strength, high drug loading capacity, and good sustained release, effectively inhibiting tumor growth and metastasis.^296^ Moreover, a novel magnetic mesoporous embolic microsphere capable of simultaneously loading doxorubicin, blocking blood vessels, and achieving MRI has been explored, showing great potential in TACE treatment with excellent drug loading, embolization, and imaging performance.^297^ Secondly, due to tumor hypoxia in the TME after TACE, tumor progression in TACE-treated patients is common. High-throughput sequencing of different models has revealed that TACE induces S100A9 through the hypoxia-inducible factor 1α-mediated pathway, leading to mitochondrial fission and ROSs production, thereby promoting metastasis. Thus, targeting S100A9 may be a promising therapeutic strategy.^298^ Additionally, embolic agents loaded with melatonin have been observed to effectively suppress tumor growth and metastasis after TACE by targeting the hypoxic TME, being safe, effective, and potentially applicable in clinical translation for TACE therapy.^299^ Furthermore, radiofrequency ablation (RFA) is also recommended by guidelines as one of the curative treatment methods for early liver tumors.^300^ However, inadequate RFA leading to intrahepatic and distant metastasis is a major cause of treatment failure. Research has shown that sublethal heat stress induced by insufficient RFA via the m6A-YTHDF1-EGFR axis has been confirmed to result in poor prognosis, thus targeting the m6A mechanism in combination with EGFR inhibitors is advantageous for suppressing HCC metastasis after RFA.^301^ Moreover, it has been demonstrated that after insufficient RFA, ICAM-1 can activate platelets via VE-cadherin and promote endothelial permeability; antiplatelet and anti-ICAM-1 therapies can be used to prevent progression of HCC after insufficient RFA.^302^ Additionally, for recurrent pulmonary metastatic diseases, multifocal metastases, or metastases located deep within the lungs, requiring extensive parenchymal resection or for patients with increased surgical risk due to comorbidities, percutaneous cryoablation is a safe and effective method.^303^

### Radiotherapy

Radiation therapy also plays a significant role in the treatment of metastatic tumors. In patients with mCSPC, bone-targeted α-emitting radioisotopes have been shown to significantly improve OS compared to standard treatment.^304^ Additionally, persistent PSA is an adverse prognostic factor for recurrence after radical prostatectomy. Salvage radiation therapy has been found to confer survival benefits to some patients.^305^ In the case of brain metastases, whole-brain radiation therapy (WBRT) is a mainstay of treatment for many patients. However, given concerns about preventing disease progression and associated toxicity, the clinical impact of WBRT has been questioned. Research has found that activation of the S100A9–RAGE–NF-κB–JunB pathway in brain metastases may mediate resistance to this treatment, with levels of endogenous S100A9 in brain lesions correlating with clinical response to WBRT. Thus, the detection of S100A9 levels in the blood could assist in personalized selection of WBRT.^306^ Due to concerns about WBRT-related toxicity, clinical practice has increasingly adopted stereotactic radiation therapy (SRT), a strategy that delivers high-dose radiation specifically to metastatic lesions. In (NSCLC brain metastases, SRT has been widely used, while WBRT remains the standard treatment for small cell lung cancer (SCLC) brain metastases. However, a clinical trial has shown that after receiving SRT, there was no significant increase in neurological death rate, leptomeningeal disease, CNS progression lesion number, or adverse events post-SRT compared to matched NSCLC. This analysis provides reliable data support for personalized decision-making in SCLC patients based on clinical expectations.^307^ In solid tumor leptomeningeal metastases, due to metastases spreading throughout the entire central nervous system (CNS) compartment, standard radiation therapy (such as WBRT) cannot halt the progression of LM along the entire neuraxis. Considering the potential toxic effects, proton whole-brain spinal cord irradiation across the entire CNS has shown better CNS PFS and better efficacy.^308^ Recent studies have found that lower serum concentrations of specific apolipoproteins (ApoE, ApoA1, and ApoJ) and higher levels of Aβ1-42 may be associated with cognitive decline following postoperative radiation therapy for brain metastases. Therefore, consultation regarding radiation-related neurocognitive decline should be provided to patients with these serum biomarkers before treatment. For patients at high risk of neurocognitive decline based on baseline biomarkers regardless of the radiation therapy regimen, consideration should be given to WBRT to reduce the risk of intracranial recurrence.^309^ The “brain radiation prediction score” constructed based on DNA features associated with recurrence risk may help quantify the individual risk of local recurrence after receiving brain-directed radiation therapy.^310^

In metastatic PCa cases with non-regional lymph node metastases or fewer than three bone metastases, and without visceral metastases, the addition of prostate radiotherapy to standard systemic therapy is linked to improved OS and PFS.^311^ In patients with newly diagnosed metastatic nasopharyngeal carcinoma, combined chemotherapy and local radiotherapy significantly improve OS compared to chemotherapy alone.^312^ Furthermore, radiation therapy has been shown to enhance systemic antitumor responses to immunotherapy. For example, adding radiation therapy to pembrolizumab significantly improves response rates to unirradiated lesions, resulting in significantly increased OS and PFS and being considered a treatment option for patients with metastatic NSCLC^313^ In a PDAC mouse model with high metastatic risk, a combination therapy of RT and PD-1 targeted IL-2 variant cytokines induces significant systemic memory immune responses, and repeated administration in mice achieving complete remission leads to sustained antitumor effects.^314^ Additionally, research suggests that surgically treated patients may experience an immunosuppressive environment characterized by hypoxia and an influx of BM cells, potentially leading to a poor response to PD-L1 blockade therapy. To address this issue, a radioimmunostimulatory nanodrug (IPI549@HMP) has been designed to target BM cells and catalyze endogenous H2O2 conversion to O2, thereby alleviating hypoxia. This finding suggests that enhanced immunogenic effects mediated by radiation therapy contribute to the reconstruction of the postoperative TME and increase sensitivity to PD-L1 therapy.^315^ In summary, radiation therapy complements other treatment modalities, significantly improves patient prognosis, and demonstrates outstanding therapeutic effects in clinical practice, providing patients with more considerable efficacy.

### Chemotherapy

Chemotherapy has been recognized as the primary treatment for stage IV CRC and unresectable metastatic patients. Studies indicate that primary tumor resection (PTR) prior to systemic chemotherapy does not correlate with prolonged OS in CRC patients with synchronous unresectable metastases. Hence, PTR should no longer be considered a standard treatment for asymptomatic primary tumors in CRC patients with synchronous unresectable metastases.^316,317^ Systemic secondary surgery combined with oxaliplatin-based intraperitoneal chemotherapy (HIPEC), compared to traditional standard monitoring, did not improve disease-free survival rates significantly. Therefore, appropriate monitoring measures may already be sufficient and effective for patients with CRC at higher risk of peritoneal metastasis.^318^ Conversely, in GC patients with peritoneal metastasis, the combination of cytoreductive surgery (CRS) and HIPEC significantly improved OS and PFS without increased morbidity or mortality rates. This suggests that CRS-HIPEC could be a potentially effective treatment for GC patients with peritoneal metastasis, especially under stringent patient selection criteria.^319^ Neoadjuvant chemotherapy (NAC) refers to chemotherapy administered before surgical tumor resection. Researchers identified highly metabolically active MRC1 + CCL18 + M2-like macrophages at metastatic sites through single-cell sequencing and demonstrated that effective NAC restored immune balance within resectable CRC liver metastases, providing evidence to design novel therapeutic combinations and undertake combination therapy in selected patients.^320^ Triple chemotherapy regimens have shown survival benefits for various advanced gastrointestinal cancers. However, attempts to intensify treatment in advanced biliary tract cancer (BTC) patients using triple-drug regimens did not show superior OS benefits compared to dual chemotherapy alone, indicating tumor-specific effects of intensified multimodal therapy; thus, CISGEM dual chemotherapy remains the frontline standard for advanced BTC.^321^ Combining chemotherapy with various treatment modalities can synergistically yield promising outcomes across different tumor types. For instance, in PD-L1-positive TNBC patients, atezolizumab combined with albumin-bound paclitaxel is an important therapeutic option for unmet treatment needs.^322^ In resectable CRC liver metastasis patients, adding perioperative systemic chemotherapy upon surgical resection increased PFS by 7%; however, perioperative treatment with cetuximab combined with chemotherapy significantly reduced PFS compared to chemotherapy alone. Therefore, cetuximab should not be considered as neoadjuvant therapy for operable CRC liver metastasis patients, underscoring the potential unforeseen consequences of molecularly targeted interventions in complex cancers.^323^ Chemotherapy is common in the treatment of metastatic patients, yet often leads to chemoresistance, a significant challenge in treating metastatic cancers. In a mouse model of BC lung metastasis, it was observed that chemotherapy-treated cancer cells secrete IL-1b, triggering the formation of NETs. These NETs can modulate neighboring cell activity by capturing and activating cytokines, thereby reducing treatment response. This reveals a potential mechanism by which chemotherapy-induced inflammation contributes to chemoresistance. Targeting the IL-1b-NET-TGF-b axis may be an effective means to reduce or prevent chemotherapy resistance in the metastatic environment.^324^ Utilizing organoid-based in vitro platforms combined with in vivo intracavity dissemination in animals aids in effectively studying the biology and drug sensitivity of clinically isolated tumor strains, potentially informing clinical treatment decisions.^325^ Additionally, chemotherapy regimens often entail systemic toxicity; for instance, oxaliplatin exhibits neurotoxicity, which can compromise patient tolerance and treatment continuity. To address this issue, researchers, based on the results of the OPTIMOX trial, introduced maintenance therapy with fluorouracil into the induction therapy regimen with fluorouracil and oxaliplatin. Subsequent randomized trials demonstrated that adding panitumumab to maintenance therapy further improves patient PFS.^326^

### Targeted therapy

Currently, chemotherapy, as the primary modality for cancer treatment, although capable of killing sensitive cells, imposes significant systemic toxicity on patients and may stimulate the proliferation of multidrug-resistant cancer cells within tumor tissues. Therefore, targeted therapy holds promise in addressing this limitation. TNBC often accompanies p53 inactivation, contributing to its increased invasiveness and therapeutic resistance. Researchers have designed a PROTAC that selectively targets MDM2 within p53-mutated/deficient TNBC cells, inducing proteasome-mediated degradation through high-affinity binding and VHL recruitment, thereby promoting TC apoptosis without toxicity to normal cells and significantly extending survival, offering an innovative potential therapeutic strategy for TNBC.^327^ In EGFR-mutated lung cancer brain metastasis models, upregulation of S100A9 in cancer cells activates the retinoic acid signaling pathway by upregulating ALDH1A1 expression, driving fatal brain recurrence. Targeting this pathway with pan-RAR antagonists may impede cancer progression and prolong patient survival.^328^ Similarly, EGFR-mutated NSCLC patients develop resistance to EGFR tyrosine kinase inhibitors (TKIs), where upregulation of CD70 in resistant cells is considered an early event in tumor resistance evolution, suggesting CD70 as a therapeutic target for acquired EGFR TKI resistance in EGFR-mutated tumors.^329^ In recent years, HER2-targeted therapies such as pertuzumab and trastuzumab have made significant strides in improving the prognosis of various cancer types. Recent clinical trials have reaffirmed the encouraging activity and durable responses of these treatments in HER2-positive metastatic BTC patients, with OS and tolerability superior to conventional second-line chemotherapy regimens, offering a new treatment paradigm for refractory metastatic BTC.^330^

**Fig. 7:**
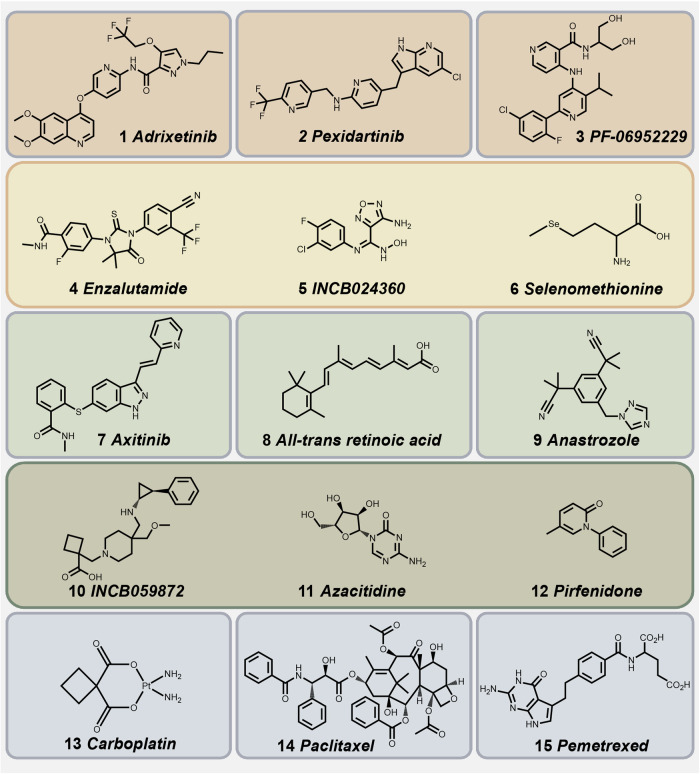
Chemical structures of small-molecule inhibitors discussed in the review. In this figure, we provide a comprehensive summary of the chemical structures of various small molecule inhibitors discussed in the review. These inhibitors are arranged in the figure according to the sequence in which they appear in the text

An advantageous approach involves using liposomal drug delivery systems and actively targeted nanotechnology, which provide the benefit of specifically targeting cancer cells.^331^ For instance, since CSCs are typically found in hypoxic tumor regions and are highly tumorigenic and chemoresistant, nanoparticles loaded with all-trans retinoic acid, camptothecin, and differentiation inducers can specifically target and eliminate CSCs in malignancies. This strategy reduces stemness-related drug resistance and prevents postoperative tumor recurrence and metastasis.^332^ Additionally, actively targeting the primary constituents within PMNs is advantageous for suppressing the process of tumor metastasis. Research has demonstrated that targeting MDSCs through a sponge-like neutrophil membrane-coated nano-system or a tumor-targeting C-peptide modified low molecular-weight heparin-all-trans-retinoic-acid micellar nanoparticle in reducing lung vascular permeability and inhibiting the implantation of CTCs, thereby suppressing PMN formation and consequently impeding lung metastasis.^333,334^ the in-situ assembled peptide nano-blanket, in addition to participating in the continuous recruitment of BMDCs, can also inhibit the activation of fibroblasts. This inhibition impedes the remodeling of the host stromal tissue that supports metastasis, reversing vascular instability and angiogenesis.^335^ Lung collagen, a significant ECM constituent, fosters a conducive milieu for CTC colonization. Tailoring a lung-targeted liposomal nanoparticle for miR-29a-3p delivery, mimicking exosomal mechanisms, achieves substantial in vivo reduction in type I collagen secretion by pulmonary fibroblasts, thus attenuating the formation of pulmonary PMN.^336^ Similarly, the EMT process and platelets are pivotal in tumorigenesis, PMN formation, and metastasis. Therapeutic strategies targeting key receptors mediating EMT stimulated by cancer-associated fibroblasts, tumor-associated adipocytes, TAMs, and MDSCs, as well as those facilitating platelet aggregation, leukocyte adhesion, and EC activation, have been developed and show promise in cancer therapy.^337,338^ A range of therapies that target multiple components present in the TME have been developed and are being evaluated, with some being tested in clinical trials. In this review, several types of intervention are summarized, mainly including small-molecule inhibitors, monoclonal antibodies and some chemical substances (Table 2), and the chemical structures of the small-molecule inhibitors mentioned have been depicted in Fig. 7.

**Table 2:** Current clinical trials testing the modified therapeutic strategies mentioned in the review

Types		Conditions	Interventions	Phase	Status	Identifier
Small-molecule inhibitors	CSF1R inhibitors	Esophageal cancer Gastric cancer Hepatocellular cancer Cervical cancer	Q702 Pembrolizumab	Phase 1b/2	Recruiting	NCT05438420
		Metastatic/advanced pancreatic or colorectal cancers	Pexidartinib Durvalumab	Phase 1	Completed	NCT02777710
	TGF-β inhibitors	Breast neoplasms Prostate neoplasms	PF-06952229 Enzalutamide	Phase 1	Terminated	NCT03685591
Monoclonal antibodies		Breast cancer Lung cancer Hepatocellular cancer Colorectal cancer Pancreatic cancer Renal cancer	NIS793 PDR001	Phase 1/1b	Completed	NCT02947165
		Advanced solid tumors	Livmoniplimab Budigalimab	Phase 1	Recruiting	NCT03821935
	CD40 mAbs	Pancreatic ductal adenocarcinoma	Mitazalimab mFOLFIRINOX	Phase 1b/2	Active, not recruiting	NCT04888312
			Odetiglucan CDX-1140	Phase 1b	Recruiting	NCT05484011
		Non-small cell lung cancer Metastatic melanoma	APX005M Nivolumab	Phase 1/2	Completed	NCT03123783
		Triple negative breast cancer	PLD chemotherapy CDX-1140 CDX-301	Phase 1	Recruiting	NCT05029999
		Ovarian cancer	CDX-1140 Bevacizumab Pembrolizumab	Phase 2	Not yet recruiting	NCT05231122
	Anti-GITR mAbs	Malignant melanoma	TRX518	Phase 1	Completed	NCT01239134
		Head and neck squamous cell carcinoma	INCAGN01876 INCMGA00012 DPV-001	Phase 1b	Recruiting	NCT04470024
		Glioblastoma	Nivolumab MK-4166 INCB024360 Ipilimumab	Phase 1	Terminated	NCT03707457
	Anti-IL-6 mAbs	Prostatic neoplasms	CNTO 328 Docetaxel	Phase 1	Completed	NCT00401765
		Pancreatic cancer	Siltuximab Spartalizumab	Phase 1b/2	Active, not recruiting	NCT04191421
Chemical substances	SLM	Clear cell renal cell carcinoma	SLM Axitinib Pembrolizumab	Phase 1/2	Recruiting	NCT05363631
	ATRA	Breast neoplasm female	ATRA Anastrozole	Phase 2	Recruiting	NCT04113863
		Acute myeloid leukemia	INCB059872 ATRA Azacitidine	Phase 1/2	Terminated	NCT02712905
	Ginsenoside Rg3	Hepatocellular Carcinoma	Anti-angiogenic Targeted Drugs Ginsenoside Rg3 TACE	Not Applicable	Not yet recruiting	NCT04523467
		Advanced gastric cancer	Ginsenoside Rg3 First-line Chemotherapy	Phase 2	Unknown	NCT01757366
		Stage I and Stage II hepatocellular carcinoma	Ginsenoside Rg3 Placebo	Not Applicable	Completed	NCT01717066
	Anti-fibrotic agents	Non-small cell lung cancer	Pirfenidone Carboplatin Paclitaxel Pemetrexed	Phase 1/1b	Active, not recruiting	NCT03177291

### Immunotherapy

**Fig. 8:**
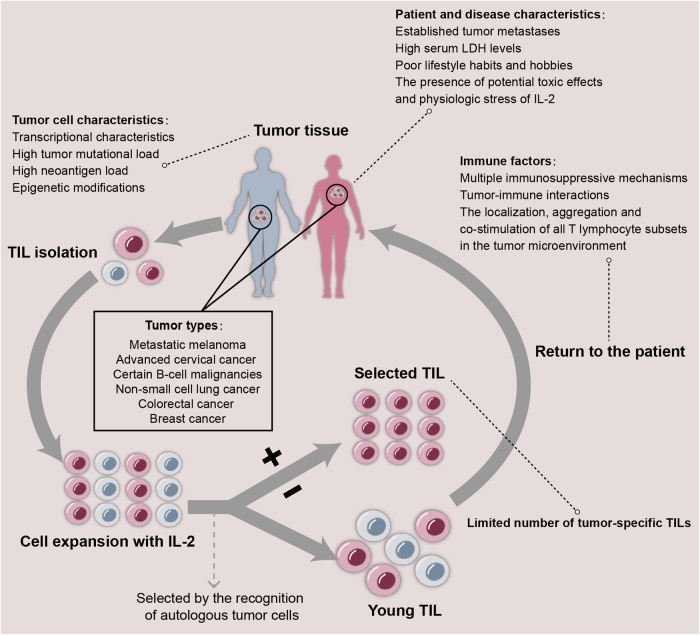
Schematic illustration of the production process and influencing factors for TIL therapy. TIL therapy is a personalized treatment of cancer. After the patient’s tumor was removed, autologous T lymphocytes obtained directly from the surgically removed tumor were then cultured and expanded under IL-2 stimulation. The amplified TILs are then selected for recognition of autologous tumor cells and the resulting product is injected back into the patient, or directly by the Young TIL method, a method that does not require in vitro selection for tumor reactivity, and the TIL is rapidly amplified and injected back into the patient. Today, TIL therapy has shown remarkable clinical results in metastatic melanoma, advanced cervical cancer, and certain B-cell malignancies. Initial efficacy has also been achieved in NSCLC, CRC, and BC. However, patient and disease characteristics such as tumor metastasis, elevated serum lactate dehydrogenase levels, or unhealthy lifestyle habits, along with potential toxicities of IL-2 and physiological stress, limit its selective use, rendering patients with severe organ dysfunction, advanced age, or frailty ineligible for treatment. Moreover, despite achieving substantial objective responses, challenges include the polyclonal nature of TIL products with only a small subset being tumor-specific, as well as immune inhibitory mechanisms, which hinder effective tumor infiltration or full exploitation of TIL anti-tumor functions. Additionally, the association between tumor transcriptional characteristics, high tumor mutational load, neoantigen load and epigenetic modifications, and poor responsiveness to TIL treatment cannot be underestimated. Meanwhile, the experimental observation suggests the complexity of the underlying tumor-immune interactions and their importance in the TIL treatment process. Thus, the localization, aggregation, interaction with tumor cells and co-stimulation of all T lymphocyte subsets in the TME are necessary for a successful antitumor immune response

Targeted therapies in clinical practice are rapidly evolving, primarily focusing on genomic alterations. Transcriptomic analysis provides an opportunity to dissect the TME. Studies have found that TME subtypes correlate with patient responses to various cancer immunotherapies, with patients having an immune-favorable TME subtype benefiting the most from immunotherapy.^339^ According to experimental research, TAMs isolated from BC can drive the positive regulatory circuit created between cancer cells and TAMs with the accompaniment of CSF1 and TNF-α, which in turn can up-regulate SIGLEC1, predicting a dismal prognosis for the patient.^340^ Also, it was found that combination therapy with the immunostimulatory effect of low-dose cyclophosphamide coupled with pharmacological inhibitors of TAMs using anti-CSF1R antibodies was effective in several highly aggressive tumor metastasis models.^341^ Simultaneously, harnessing the superior ability of KCs to capture circulating bacteria, a single administration of detoxified Escherichia coli engineered to produce clustered regularly interspaced short palindromic repeats associated with CasΦ (CRISPR/CasΦ) effectively edits genes of interest in KCs. This approach can overcome KC functional impairments and yields significant therapeutic effects on various types of murine metastatic liver cancer.^342^ These further suggest that targeting TAMs may be therapeutically beneficial and may improve immunotherapeutic efficacy by selecting the right combination of immunotherapies. To date, T cells have been a primary focus in immunotherapy research. For example, it has been observed that the injection of agonistic monoclonal antibodies targeting GITR, a costimulatory receptor on Treg cells, can induce tumor regression by enhancing T cell function and reducing the suppressive capacity of Tregs. At the same time, the combination of anti-Gal-9 antibodies produced synergistic antitumor activity.^343^ Autologous T cells that have been surgically taken from the patient’s own tumors, after a phase of culture expansion stimulated by IL-2, are eventually reintroduced into the patient. Following adoptive cell infusion, recognition triggered by neoantigens and T-cell-mediated killing both aid in the removal of tumors.^344^ (Fig. 8) Nonetheless, tumor-infiltrating B lymphocytes, including both B cells and plasma cells, appear to perform an indispensable synergistic contribution to preventing tumors from growing and have also demonstrated significant predictive and prognostic value in a variety of malignancies.^345^ Metabolites in TME promote the aberrant accumulation of Treg in tumors by supporting metabolic reprogramming of Tregs and interfering with metabolic regulatory circuits formed by effector T cells, which in turn promote the formation of immunosuppressive TME.^346^ At the same time, metabolic pre-treatment can improve the efficacy of pericyte immunotherapy, and targeting metabolic parameters during immune checkpoint blockade treatment can produce therapeutic synergy.^347^ Therefore, a holistic understanding of immune cell metabolism can facilitate the development of rational and effective immunotherapy strategies. Modified strategies via targeting multiple components in TME to modulate therapeutic resistance are summarized in Table 3.

**Table 3:** Examples of strategies targeting certain pathways as therapy resistance modulators

Targets	Cancer types	Related drugs	Mechanism	Refs.
TAMs	Breast cancer	CTX, CSF1R inhibitors	Immunostimulatory effects of CTX combined with small molecule CSF1R inhibitors can induce tumor regression and the expansion of polyclonal long-lived central memory T cells and activated B cells within TLS.	^340,341^
	Pancreatic cancer, Mesothelioma, Melanoma	CD40 mAb, chemotherapeutic drug	Destruction of stroma by CD40-activated macrophages can enhance chemotherapy delivery.	^386^
Tregs	Melanoma, Renal cell carcinoma, Breast cancer	agonistic anti-GITR mAbs, anti-CTLA-4 mAbs, anti-Gal-9, PD1 antibody	1) GITR costimulation attenuates T reg–mediated suppression or improves CD4+ and CD8+ effector T cells. 2) Anti-Gal-9 therapy expands intratumoral TIM-3+ cytotoxic CD8 + T cells and Treg cells.	^343,346,387^
TIL-Bs	Squamous cell carcinoma	anti-PD-L1 antibody	The addition of an anti-PD-L1 antibody to radiation therapy converted a Breg cell response to an effector B cell response associated with improved tumor control.	^388^
	Glioblastoma	agonistic anti-CD40 antibody, anti-PD-L1 antibody	BVax migrates to key secondary lymphoid organs and is proficient at antigen cross-presentation, which promotes both the survival and the functionality of CD8 + T cells.	^389^
Cancer cells	Lung cancer, Glioblastoma, Pancreatic cancer	selenomethionine, methionase, Annexin-V	FP catalyzes the conversion of selenomethionine to toxic methylselenol, thereby preventing methionine supplementation to cancer cells.	^390^
CSCs	Breast cancer	differentiation-inducing agent, ATRA, camptothecin	The controlled release of drugs in CSCs and the targeted mediating of the death process of CSCs are conducive to reducing dry-related drug resistance.	^332,391^
MDSCs	Breast cancer	redox-responsive polymer, doxorubicin	PPcDG/D nanoparticles coating with neutrophils membrane showed obvious natural tropism to postoperative inflammatory site and inhibited the recruitment and functions of MDSCs.	^333^
		COS-ATRA, doxorubicin	Both COS and ATRA blocked NF-κB inflammatory signaling pathway in tumor and MDSCs. ATRA also depleted MDSCs in lungs and tumors, thereby regulating the immunosuppressive microenvironment.	^334^
Fibroblasts	Lung metastasis	therapeutic miR-29	Lung-targeting liposomal nanovesicle (DOTAP/cholesterol–miRNAs to 4:1) carry miR-29a-3p and mimic the exosomes, which down-regulated collagen I secretion by lung fibroblasts and impeded the remodeling of the host stromal tissue.	^336^
Epithelial- Mesenchymal Transition	Breast cancer	CCT365623	CCT365623, a pharmacological inhibitor of LOX, which disrupts EGFR cell surface retention and reversed LOX-induced EMT and significantly decreased the invasive ability.	^337,392–394^
		SB431542, pirfenidone	Inhibitors targeting TGF-β signaling are shown to abrogate CAFs-induced EMT in breast cancer cells, which delays the growth of primary and metastatic tumor cells.	
		PEG-LPrA2	PEG-LPrA2, acting as a leptin receptor antagonist, significantly reduced tumor growth in breast cancer by repressing ERK, AKT or VEGF upregulation.	
		ginsenoside Rg3	Ginsenoside Rg3 blocked MDSC-mediated EMT and stemness acquisition in cancer cells, resulting in tumor suppression.	
PLTs	Ovarian cancer	siltuximab, paclitaxel	Blocking IL-6 and TPO production contributes to reducing platelet counts or platelet activation, which might attenuate cancer progression.	^338^

With advances in tumor immunology and nanotechnology, therapeutic cancer vaccines have garnered significant attention, aiming to enhance tumor-specific T-cell immune responses. Experimental observations show that intravenously injected cancer vaccines can activate antigen-specific CD8+ T cells and promote tumor regression through TME modulation dependent on type I IFN. This finding suggests that the generation of tumor-specific CD8+ T cells combined with TME remodeling constitutes a promising strategy for driving the success of tumor immunotherapy.^348^ However, the lack of sustainable immune activity post-vaccination limits its long-term efficacy. In this regard, researchers have developed a bisphosphonate nano-vaccine system in combination with RFA, which, through alternately induced immune responses, prolongs the duration of anti-tumor immune responses, inhibiting the recurrence and metastasis of CRC liver metastases, providing direction for sustainable regulation and precise delivery of cancer vaccines.^349^ Furthermore, chimeric antigen receptor (CAR) T cells have shown unprecedented responses in subgroups of refractory patients with B cell and plasma cell malignancies, sparking widespread research interest in academia. Preliminary preclinical and clinical results indicate that CAR-T cells targeting B7-H3 and GD2 may have potential therapeutic benefits in treating CNS malignancies.^350,351^ In various models of metastatic medulloblastoma and PFA ependymoma in mice, infusion of these CAR-T cells alone or in combination with azacitidine into the cerebrospinal fluid has shown efficient therapeutic responses, laying the theoretical foundation for translating these methods into clinical trials in humans.^351^ It is anticipated that the next generation of CAR-T cells will incorporate advancements in genetic engineering and synthetic biology to improve functionality and persistence while minimizing treatment-related toxicities. These strategies, combined with various allogeneic cell therapies targeting the immunosuppressive TME, are expected to expand the impact of CAR-T cell therapy in the field of oncology.^352^

### Endocrine therapy

Endocrine therapy (ET) is an effective treatment for hormone receptor-positive tumors, primarily intervening in the endocrine system using hormones or anti-hormonal agents to inhibit or block TC growth and spread. Mainly applied in hormone-dependent tumors such as breast and PCas, it includes various drugs: selective estrogen receptor (ER) modulators, aromatase inhibitors, non-steroidal agents, and steroidal agents.^353,354^ ER and progesterone receptor (PR) signaling pathways regulate breast development and influence BC occurrence. However, while ER is a established driver in ER+ disease, PR’s role remains contentious. Studies suggest that the effects of progesterone may vary depending on the expression of ER, PR, and other molecular markers in patients. Thus, the efficacy of progesterone therapy may be patient-specific, emphasizing the importance of personalized treatment strategies in ET.^355^ In ER + BC, ET remains a primary treatment modality. Mutations in the ESR1 gene encoding ER have been reported to be closely associated with ET resistance, prevalent in newly diagnosed ER + BC metastatic and locally recurrent cases, and associated with shorter PFS. This underscores the importance of early detection of ESR1 mutations in metastatic and recurrent settings, potentially guiding patient management, follow-up, and treatment planning.^356^ Clinical trial results have demonstrated the potential efficacy of lasofoxifene as a treatment for late-stage or metastatic ER + BC in women expressing constitutively active ERα mutations. Combining lasofoxifene with Palbociclib effectively inhibits tumor growth, enhancing the efficacy of ET.^357^ However, resistance to ET and cyclin-dependent kinase 4/6 inhibitors (CDKIs) is nearly inevitable in most ER + BC patients. Through genomic and metabolomic analyses of tumors, a subset of metastatic ER + BC highly dependent on oxidative phosphorylation has been identified. Thus, oxidative phosphorylation represents a promising target for resistant ER + BC patients to endocrine and Palbociclib therapy.^358^ Activation of the PI3K/AKT-mTOR pathway has also been shown to be a mechanism of early adaptive resistance to CDKIs.^359^ Therefore, developing triple therapies involving fulvestrant, CDKIs, and AKT inhibitors may help reverse tumor progression, particularly in tumors showing high levels of p-AKT.^360^ Additionally, enobosarm treatment in women with ER+, HER2-negative, and AR-positive disease intolerant to ET offers clinical benefits and prolonged PFS with low, manageable adverse events, supporting further clinical research into selective AR activation strategies for such endocrine-resistant BC.^361^ Transcriptional analysis of ET combined with CDKI-resistant cell lines reveals upregulation of Polo-like kinase 1 (PLK1), and PLK1 inhibition leads to significant regression of highly proliferative CCND1-driven tumors, correlating with extended metastasis-free survival. Thus, PLK1 inhibitors hold clinical utility in late-stage BC patients driven by CCND1.^362^ PCa develops resistance to androgen deprivation through adaptive upregulation of the AR in a low testosterone microenvironment. Bipolar androgen therapy disrupts this adaptive regulation in CRPC, rendering it sensitive to subsequent anti-androgen therapies with meaningful clinical activity and safety.^363^ Additionally, the combination of apalutamide and abiraterone plus prednisone delays resistance development by dual inhibition of the androgen signaling axis, improving outcomes in mCSPC patients.^364^

However, there are exceptions to the systemic use of ET as initial treatment, such as compromised overall health and/or life-threatening conditions. Studies have found that 15%–50% of hormone receptor-positive, HER2-negative metastatic BC patients receive chemotherapy as first-line therapy.^365^ Therefore, optimizing patient survival and quality of life by selecting the most beneficial treatment modality as the primary approach is crucial. In this context, CTC counts may be considered a reliable biomarker for guiding the choice between chemotherapy and ET.^366^ It’s noteworthy that besides efficacy and safety, the impact of treatment on quality of life is also essential when deciding the therapeutic approach for metastatic BC patients. Research indicates that compared to capecitabine, patients treated with palbociclib/ET experience significantly delayed deterioration in global health status/quality of life and various functional and symptom scales, further demonstrating the tolerability advantage of palbociclib/ET treatment and supporting its role as a proactive treatment choice.^367,368^ Additionally, incorporating CDKIs into ET regimens may provide varying benefits depending on a patient’s level of endocrine sensitivity, which is critical for clinical decision-making regarding the risk-benefit ratio of using combined CDKIs and ET versus ET alone.^369^ However, it’s currently unclear whether patients receiving sole ET versus combined ET and CDKIs would yield similar outcomes, highlighting the need for further research into biomarkers predictive of CDKI treatment response. For instance, plasma thymidine kinase 1 activity has been shown to be a clinically effective novel circulating prognostic marker in ER+/HER2− metastatic BC patients receiving ET and Palbociclib treatment.^370^ Additionally, synergistic effects between hormonal therapy and radiation therapy have been established in the oncology field. For instance, in men with oligometastatic PCa, combining MDT with intermittent hormonal therapy achieves good disease control while promoting extended intervals of normal testosterone, resulting in OS benefits.^371^

## Conclusion and perspective

Advancements in understanding tumorigenesis have established that the metastatic process in TCs is organ-specific and can manifest at various stages of the disease. This process cannot be viewed as a simple concatenation of discrete steps. Complex crosstalk may exist between different stages, and certain mechanisms may span across multiple steps in the metastatic cascade. The specific molecular mechanisms that facilitate the development of an immunosuppressive state during tumor metastasis and the ability of CTCs shed from primary sites to evade immune surveillance and establish distant colonies remain largely unclear. These processes may be influenced by the diversity, activity, and quantity of immune cells within the TME and PMN. Considering the swift development of single-cell and spatial analysis technologies, it is anticipated that significant progress will be made in defining the developmental characteristics and cellular heterogeneity of multiple cells in TME, as well as the intricate crosstalk pathway.

Merely intervening at metastatic sites after macrometastasis has occurred is often inadequate; future cancer treatments should focus on the early prediction and comprehensive control of metastasis at a systemic level to improve treatment efficacy. Continuous improvements in imaging technologies and molecular detection techniques allow for the early detection of small-volume and occult metastatic foci suitable for localized therapy, facilitating timely diagnosis and intervention. Furthermore, the escalation of systemic treatments, the evolution of multimodal comprehensive therapies, and the further integration of specialized oncological care with palliative interventions hold promise for enhancing the survival rates of early-stage cancer patients, while concurrently improving PFS and quality of life for late-stage tumor patients. In conclusion, an in-depth exploration of metastasis-related mechanisms and expeditious clinical translation of trials are of paramount significance in ameliorating clinical outcomes for patients afflicted with metastatic cancers.
